# Multicomponent Chiral Quantification with Ultraviolet Circular Dichroism Spectroscopy: Ternary and Quaternary Phase Diagrams of Levetiracetam

**DOI:** 10.1021/acs.molpharmaceut.2c00825

**Published:** 2022-12-05

**Authors:** Maxime D. Charpentier, Raghunath Venkatramanan, Céline Rougeot, Tom Leyssens, Karen Johnston, Joop H. ter Horst

**Affiliations:** † EPSRC Centre for Innovative Manufacturing in Continuous Manufacturing and Crystallization (CMAC), University of Strathclyde, Technology and Innovation Centre, 99 George Street, GlasgowG1 1RD, U.K.; ‡ UCB Pharma SA, chemin du Foriest, 1420 Braine-L’Alleud, Brussels1070, Belgium; § Institute of Condensed Matter and Nanosciences, UCLouvain, Place L. Pasteur 1, Brussels1070, Belgium; ∥ Department of Chemical and Process Engineering, University of Strathclyde, James Weir Building, 75 Montrose Street, GlasgowG1 1XJ, U.K.; ⊥ Laboratoire Sciences et Méthodes Séparatives (SMS), Univ Rouen Normandie, UR 3233, F-76000Rouen, France

**Keywords:** cocrystals, phase diagrams, crystallization, chirality, pharmaceuticals, solubility

## Abstract

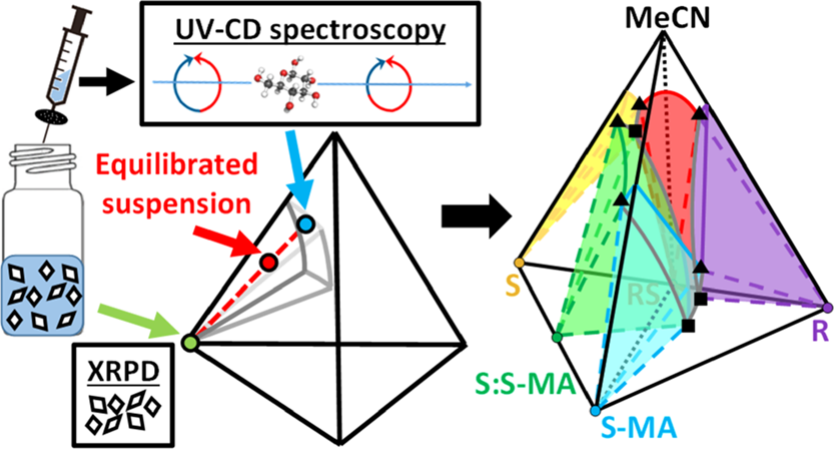

Chiral molecules are challenging for the pharmaceutical industry because although physical properties of the enantiomers are the same in achiral systems, they exhibit different effects in chiral systems, such as the human body. The separation of enantiomers is desired but complex, as enantiomers crystallize most often as racemic compounds. A technique to enable the chiral separation of racemic compounds is to create an asymmetry in the thermodynamic system by generating chiral cocrystal(s) using a chiral coformer and using the solubility differences to enable separation through crystallization from solution. However, such quaternary systems are complex and require analytical methods to quantify different chiral molecules in solution. Here, we develop a new chiral quantification method using ultraviolet-circular dichroism spectroscopy and multivariate partial least squares calibration models, to build multicomponent chiral phase diagrams. Working on the quaternary system of (*R*)- and (*S*)-2-(2-oxopyrrolidin-1-yl)butanamide enantiomers with (*S*)-mandelic acid in acetonitrile, we measure accurately the full quaternary phase diagram for the first time. By understanding the phase stabilities of the racemic compound and the enantiospecific cocrystal, the chiral resolution of levetiracetam could be designed due to a large asymmetry in overall solubility between both sides of the racemic composition. This new method offers improvements for chiral molecule quantification in complex multicomponent chiral systems and can be applied to other chiral spectroscopy techniques.

## 1 Introduction

Because of their mirror image symmetry, enantiomers exhibit the same enantiopure physical properties, such as the crystal melting point, solubility, molecular reactivity with achiral molecules, and the same response in analysis by conventional spectroscopy methods [nuclear magnetic resonance, ultraviolet (UV), and infrared].^1^ However, their interaction with chiral systems, for example, a chiral drug interacting with chiral receptors in the human body, differs and hence induces different biological activities. In many cases, one enantiomer has a desired therapeutic effect, while the other may have no effect or even a harmful effect.^2−5^ In addition, a non-active counter-enantiomer is an impurity that can constitute up to 50% of the product, which has economic consequences.^6^ This is the case for (*S*)-2-(2-oxopyrrolidin-1-yl)butanamide, known commonly as levetiracetam, a nootropic drug used as an anticonvulsant to treat epilepsy.^7^ Although the pure enantiomer product is desired for chiral drugs, the process of obtaining enantiopure active pharmaceutical ingredients (APIs), called chiral resolution, is challenging. Many chiral molecules are synthesized by non-stereoselective chemical reactions, leading to racemic mixtures that require separation. Crystallization is the preferred strategy at the industrial scale as it is relatively inexpensive^8,9^ and can be highly selective depending on the solid–liquid equilibria between enantiomers in solution.^1,10^ In 5–10% of cases, enantiomers crystallize separately to form a conglomerate, which is a physical mixture of enantiopure crystals that is amenable to chiral resolution processes.^11−21^ However, in 90–95% of cases, a racemic crystal is formed, and chiral resolution through crystallization is difficult or even impossible.^1,22,23^ An alternative resolution method is to generate multicomponent crystals. If chiral molecules can be ionized, Pasteurian resolution^24−26^ is possible by formation of diastereomeric salts with a resolution agent. Otherwise, a conglomerate of enantiopure cocrystals or solvates can emerge using an achiral coformer or a solvent.^27−29^ Finally, using a chiral coformer can either induce formation of a diastereomeric pair of enantiopure cocrystals or an enantiospecific cocrystal.^30−35^

Understanding these multicomponent systems requires the acquisition of accurate phase diagrams that are key to designing robust and reliable crystallization processes,^36,37^ especially for chiral molecule separations.^25,38^ Phase diagrams represent compositional phase domains for equilibrium states of a system. The equilibrium state is strongly dependent on the system’s intensive properties, such as temperature and overall component compositions. However, phase diagrams become more complex as the number of components increases. In the case of chiral resolution by crystallization, ternary phase diagrams are commonly used to understand the solid–liquid equilibria between enantiomers in a solvent,^32,35,39−41^ a single enantiomer with a salt-former or a coformer,^31,32,35,42^ and diastereomeric salt systems.^25,26^ However, to truly understand and optimize a chiral resolution process of a racemic compound with a chiral coformer (or salt-former) in a solvent,^31,32,35,43^ it is necessary to know the quaternary phase diagram.

Multicomponent chiral phase diagrams increase in complexity as the number of chiral components increases because of the difficulty in quantifying them. For instance, the study of two symmetrical enantiospecific cocrystals requires the quantification of four chiral molecules in a solvent to determine the phase diagrams.^44^ Therefore, accurate quantitative methods to measure the concentration of all chiral molecules and to distinguish between two enantiomers are needed. Chiral quantification methods usually involve first measuring the components’ total concentration using gravimetry,^39^ titration,^40^ UV–vis spectroscopy,^45^ or achiral high-performance liquid chromatography (HPLC)^35^ and then quantifying the enantiomer’s concentrations using polarimetry^46^ or chiral HPLC.^23,39^ Polarimetry can only characterize a single variable variation, making quantification unreliable if more than one pair of enantiomers is present,^47^ and also presents issues such as low sensitivity and influence by other components and temperature variation.^48,49^ Chiral HPLC does not have these disadvantages and is more widely used. It can quantify two enantiomers in a single step,^23,41,50^ and for non-enantiomeric chiral molecules, quantification can be designed with both achiral and chiral HPLC methods.^32,45^ However, quantification of two enantiomers and at least one other chiral molecule increases the complexity of finding chromatography separation conditions. A combination of two different methods, such as achiral and chiral HPLC, often becomes necessary.^16,35,44^ The requirement for multiple chromatography columns and mobile phases becomes a disadvantage, as new HPLC methods need to be developed for every chiral multicomponent system studied.^51^

**Figure 1:**
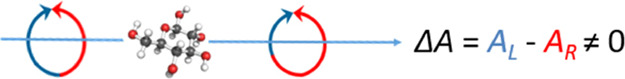
Circular dichroism: a light source composed of an equal amount of left-handed (blue) and right-handed (red) circularly polarized light, one of which is preferentially absorbed by a chiral molecule. A differential absorbance Δ*A* is measured between the absorbance of left-handed light *A*_L_ and right-handed light *A*_R_.

An interesting alternative to analyze chiral molecules is circular dichroism (CD). This technique is based on the differential interaction of a chiral molecule with left and right circularly polarized light (Figure 1) and is commonly used for structure and conformation determination of chiral molecules and proteins.^52−54^ Ultraviolet-circular dichroism (UV-CD) is CD in UV wavelengths and has proven its efficiency to quantify enantiomers in solution.^55−58^ Two signals are measured simultaneously: one is the UV signal that depends on all the molecules dissolved, and one is the CD signal that depends on the differential concentrations between the chiral compounds present. The advantage of UV-CD is that it can simultaneously detect more than one pair of enantiomers with a high sensitivity.^54,59^ The signals depend on component interactions in their spectroscopic behavior across a range of wavelengths. With the use of chemometrics^60−63^ for data analysis, complex spectra can be understood. The composition information can be linked to the spectra to develop robust calibration models allowing unknown solutions to be quantified. Indeed, chemometrics on absorption spectroscopy rely on the Beer–Lambert law,^64,65^ a proportionality relation between absorbance and concentration at every wavelength measured. Therefore, multivariate methods consider the different wavelength variables to quantify the system with improved accuracy.^66^ Previous quantification work with CD used a two-step approach with multivariate curve resolution to decompose datasets into individual component spectra and estimate their relative contributions, which are later transformed into absolute trends by fitting known values and performing a two-point calibration.^67^

In this study, we propose a new approach with multivariate partial least squares (PLS) calibration models^68,69^ to quantify chiral multicomponent systems using UV-CD spectroscopy. With this method, we determine chiral phase diagrams in the quaternary system of 2-(2-oxopyrrolidin-1-yl)butanamide enantiomers (R and S), (*S*)-mandelic acid (S-MA), and acetonitrile (MeCN) at 9 °C. This system was previously found to have stable solid phases of the pure solutes, a stable racemic compound between enantiomers, and an enantiospecific 1:1 cocrystal between S and S-MA.^31,35^ The ternary phase diagrams of this system have until now only been estimated using limited data acquired from a combination of HPLC methods.^35^ In this work, we first present a revaluation of the latter data with our method and propose a more accurate representation of the ternary phase diagrams. Then, we construct the full isothermal quaternary phase diagram for the first time, by acquiring many solubility data inside the tetrahedron plot. With the understanding of the solid phase stability and the influence of component compositions on their solubility, the chiral resolution of levetiracetam by enantioselective cocrystallization can be designed from the phase diagram data.

## 2 Experimental Methods

### 2.1 Materials

**Figure 2:**
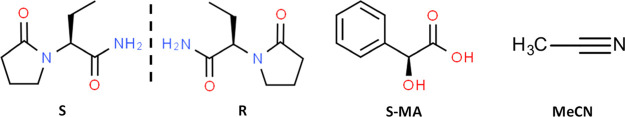
Chemical structures of the four components levetiracetam S (antiepileptic drug), its counter enantiomer R, S-mandelic acid (S-MA), and the solvent acetonitrile (MeCN).

To distinguish components in the following study, the 2-(2-oxopyrrolidin-1-yl)butanamide enantiomers will be labeled **R** and **S**, and their racemic compound **RS**, the coformer S-mandelic acid **S-MA**, the enantiospecific cocrystal **S:S-MA**, and the solvent acetonitrile **MeCN** (Figure 2). The commonly known names are levetiracetam for S and etiracetam for RS. Levetiracetam is the biologically active enantiomer and is a medication used to treat epilepsy. R, S, and RS were provided by UCB Pharma. S-MA (≥99%, Sigma-Aldrich) and MeCN (HPLC grade, 100%, VWR Chemicals) were used as received. S:S-MA was crystallized by slow evaporation of a 1:1 molar ratio solution in methanol (MeOH) and confirmed by X-ray powder diffraction (XRPD). The XRPD patterns of materials used and their references can be found in the Supplementary Information (Section S1). All solid phases present specific diffraction peak positions that permit assessment of their presence in solid mixtures.

### 2.2 X-ray Powder Diffraction

XRPD analyses were performed using a Bruker D8 Advance II diffractometer with Debye–Scherrer transmission from a Cu source radiation (1.541 Å) with an operating voltage of 40 kV, current 50 mA, Kα1 Johansson monochromator, and 1 mm anti-divergence slit. A Bruker D2 Phaser diffractometer was also used, with Bragg–Brentano reflection θ/θ geometry from a Ni filtered Cu source radiation (1.541 Å) with an operating voltage of 30 kV, current 10 mA, and 0.2 mm anti-divergence slit. A scanning range of 2θ values from 4° to 35° was applied with a 0.017° step and a step time of 1 s.

### 2.3 Ultraviolet-Circular Dichroism Spectroscopy

UV-CD spectroscopy was performed using a Chirascan-Plus spectrometer from Applied Photophysics, constantly purged with a nitrogen flow. The samples were analyzed in a Hellma quartz cell with a 0.1 mm path length. Both UV and CD spectra were collected with a 0.5 nm step and 1 second per point in the 200–260 nm range. The background of pure acetonitrile was measured and automatically subtracted from the spectra using the instrument software. As the detector is saturated when solutions with a total concentration of dissolved components exceed 5 mg/mL, the calibration range is set from 0.5 to 5 mg/mL, and all samples were diluted to fall into this calibration range. The UV and CD spectra are expressed in, respectively, absorbance units and ellipticity units (θ), a value proportional to CD. The data were collected using Chirascan Pro data V4.4.2.0, and the analysis of the UV-CD data was done using Origin Pro 2017 and Pls_toolbox 4.0 by Eigenvector research Inc. The spectra of both UV and CD were pre-processed with first derivative baseline correction followed by Savitzky–Golay smoothing^70^ of a second-order polynomial with five window points and mean centring.^71^ The spectra were otherwise free of artifacts and baseline issues, so no additional pre-processing was done.

### 2.4 Development of a Multivariate Calibration for Quantification

#### 2.4.1 Calibration Samples

**Figure 3:**
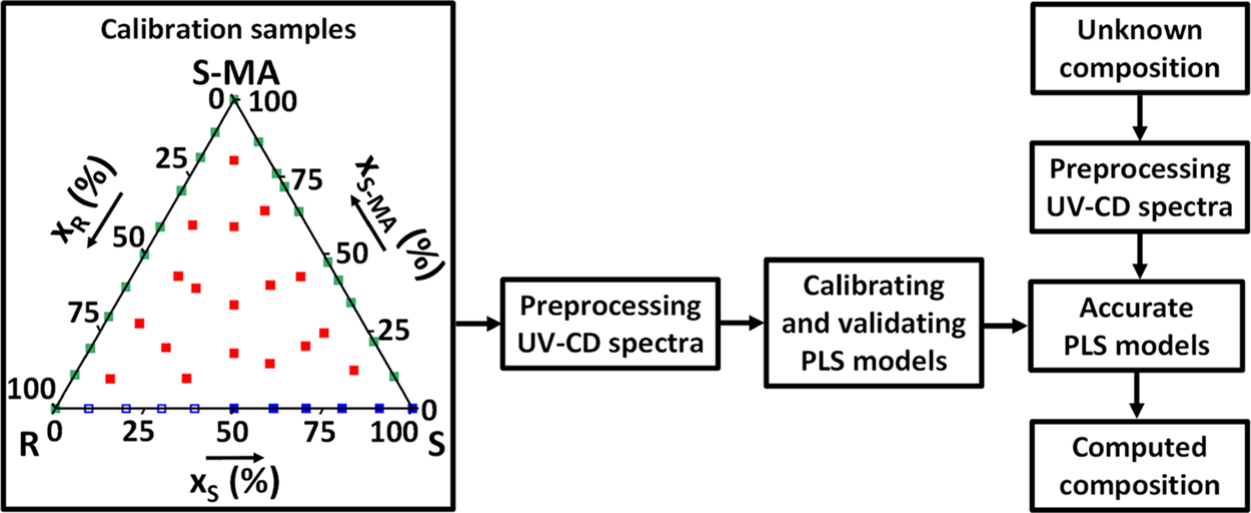
Design of the multivariate calibration, with calibration samples as input, to obtain PLS models allowing the computation of unknown compositions from their UV-CD spectra. The distribution of calibration samples is represented by their solvent free mass fraction in components. For each fraction, five solutions of varying total concentration were prepared from successive dilutions into the UV-CD calibration range (0.5–5 mg/mL), and the related UV and CD spectra were measured and gathered to build the model. Blue points correspond to the R/S/MeCN ternary section, open squares being obtained from symmetry of the experimental CD spectra with S being in excess. Green points correspond to ternary sections S/S-MA/MeCN and R/S-MA/MeCN. Red points correspond to quaternary compositions containing R, S, and S-MA in MeCN.

A multivariate calibration model using samples of known composition, i.e., calibration samples, was developed to allow the measurement of unknown composition solutions from UV-CD spectra. The chosen independent variables in the 4-component calibration samples are the mass fraction *x*, for R (*x*_R_), S (*x*_S_), and S-MA (*x*_S-MA_), with the solvent MeCN mass fraction *x*_MeCN_ = 1 – *x*_R_ – *x*_S_ – *x*_S-MA_. The construction of the model was to allow quantification of equilibrated samples from a quaternary phase diagram, which is a tetrahedron plot whose triangular faces are isothermal ternary phase diagrams. The calibration space, therefore, was designed to cover the entire quaternary space, consisting of the perimeter and the interior of the tetrahedron. Experimental solvent free component ratios of the calibration samples are shown in Figure 3. Each ratio (square) represents five calibration samples prepared by successive dilutions of the same bulk solution within the UV-CD calibration range of the molecules (0.5 to 5 mg/mL total concentration), allowing the total concentration for all components to be covered accurately. This calibration sample preparation method yields a calibration data set with 270 compositions. The 270 calibration sample compositions can be found in the Supplementary Information (Table S1). For each calibration sample, UV and CD spectra were measured.

#### 2.4.2 Design of Multivariate Partial Least Squares Calibration Models

The modeling for quantitative determination of *x*_R_, *x*_S_, and *x*_S-MA_ in unknown solutions, using experimental UV and CD spectra (Figure 3), requires a calibration using UV and CD spectra of the calibration samples. Here, we use a multivariate PLS calibration.^68,69^ Two calibration models were designed, one for the UV data and the other for CD data. Both types of signals are influenced differently by the concentration in all dissolved components (R, S, or S-MA). They both follow the Beer–Lambert proportionality law^64,65^ between absorbance and concentration at every wavelength measured. For UV spectra, because R and S absorb UV identically, two variables were defined as influencing the signal in the calibration: the total enantiomer mass fraction *x*_S+R_ = *x*_S_ + *x*_R_ and the S-MA mass fraction *x*_S-MA_. However, for CD, the two enantiomers R and S have a symmetrical response and the spectra depend on the differential mass fraction *x*_S–R_ = *x*_S_ – *x*_R_ between enantiomers. Therefore, two variables were defined as influencing the CD spectra in the calibration: the differential mass fraction between enantiomers *x*_S–R_ and the mass fraction in S-MA *x*_S-MA_. With *x*_S+R_ and *x*_S–R_ from UV and CD data, the enantiomeric excess
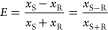
was computed and *x*_R_ and *x*_S_ were retrieved as
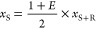
and
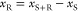


Since only one enantiomer of mandelic acid (S-MA) is present, both UV and CD calibration models yield the total S-MA concentration *x*_S-MA_.

After their acquisition, all spectra were pre-processed (Figure 3) by first derivative baseline correction followed by the Savitzky–Golay smoothing^70^ of the second-order polynomial with five window points and mean centering.^71^ This maintains the shape of the spectra and allows separation between the peaks and removal of artifacts, such as baseline shifts or noise,^66^ thus improving the predictive performance of the calibration models. The pre-processed data of both CD and UV were partitioned into a calibration (80%) and a validation (20%) dataset using the Kennard–Stone algorithm,^72^ which provides a representative split that gives a uniform distribution of samples.

Finally, the multivariate calibration models were built using PLS regression^68,69^ to relate the spectral data to the compositions *x*_S+R_, *x*_S–R_, and *x*_S-MA_. PLS is a multivariate regression method with compression of spectral data beforehand to reduce the number of variables present.^69,73^ The compressed variables obtained in PLS are referred to as latent variables (LVs). The models were validated internally and externally using cross-validation and validation datasets to test their reliability and accuracy.^60^ To minimize overfitting, the optimum LVs were chosen with a maximum explained variance for cross-validation using a random subset approach with 30 data splits and 15 iterations.

To compare the model’s predictions with experimental results from another quantification method, 28 compositions of different ratios in S and S-MA were analyzed simultaneously by UV-CD spectroscopy and the gravimetric method, i.e., measuring solubility by the mass difference between a solution and its solid obtained after complete evaporation.

### 2.5 Phase Diagram Construction: Equilibration Technique

**Figure 4:**
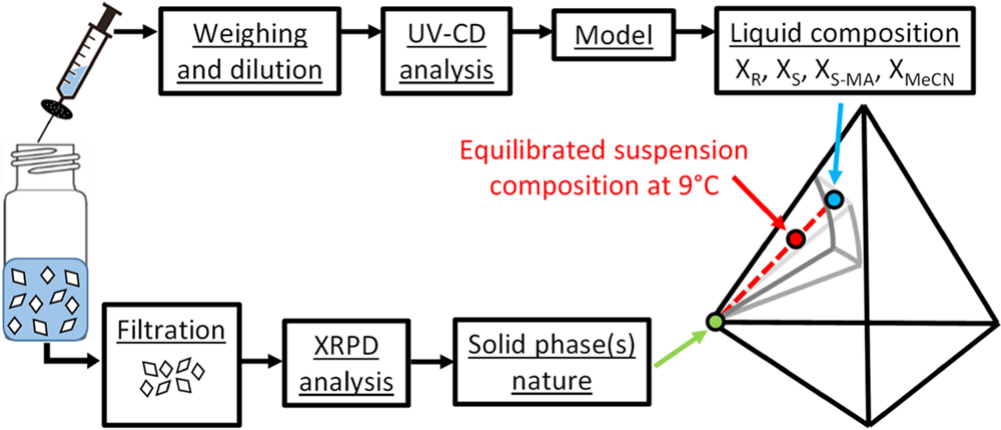
Protocol to obtain phase diagram composition from isothermal suspension at 9 °C after 14 days.

The experimental compositions for equilibration were estimated at the chosen temperature of 9 °C for phase diagram construction, based on existing data.^35^ These compositions were prepared in 2 mL sealed vials. After dissolution at 50 °C, they were cooled down to 9 °C and seeded with stable solid phases in the corresponding system, to form stable suspensions. All vials were stored isothermally at 9 ± 1 °C under stirring, using a Polar Bear Plus apparatus (Cambridge Reactor Design, UK) that enabled simultaneous equilibration of batches of 28 compositions. The compositions were left to equilibrate for 14 days after which the saturated solution and solid compositions were determined, which led to phase diagram points as summarized in Figure 4. The saturated liquid phases were sampled using a syringe with a filter. To obtain a final solution whose total component concentration is in the UV-CD calibration range for that system, a sample dilution ratio (i.e., the total mass of the dilution solvent divided by the mass of the saturated solution sample) from 10 to 300 was applied depending on the phase diagram region. Due to high dilution ratios, the liquid properties between saturated liquids and diluted samples vary a lot. Therefore, weighing of saturated liquid and added solvent was mandatory for precision, as working with volumes proved to induce a significant error in data. The diluted solutions were then analyzed by UV-CD spectroscopy. The obtained spectra were pre-processed and used as input into the model to determine the mass fractions *x*_R_, *x*_S_, *x*_S-MA_, and *x*_MeCN_ of each component in the diluted solution. The saturated liquid composition for each sample was then computed using the calculated sample dilution ratio and converted to molar fraction *X* to position the experimental point in the phase diagram. The solid phases in equilibrium with the saturated liquid were analyzed by XRPD after filtration of the suspensions to conclude on the phase diagram region the point belongs to. Eutectic points and quaternary points, corresponding to solutions equilibrated with more than one solid, are identified by XRPD in which more than one solid phase is measured. When not measured experimentally, they are estimated at the intersection of extrapolated neighboring solubility curves/surfaces.

## 3 Results

**Figure 5:**
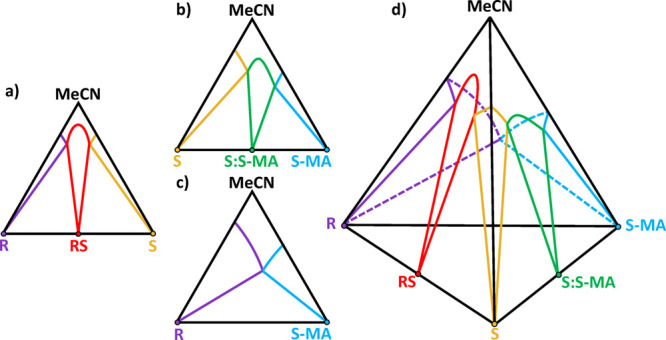
(a–d) Isothermal and isobaric schematic phase diagrams of the four-component system: (*R*)- and (*S*)-2-(2-oxopyrrolidin-1-yl)butanamide (R and S), (*S*)-mandelic acid (S-MA), and the solvent acetonitrile (MeCN). The ternary phase diagrams were estimated in a previous study,^35^ while solubility measurements inside the tetrahedron are tackled for the first time in the present study to understand phase stability and solubility variations in the quaternary diagram.

Using the UV-CD spectroscopy data from calibration samples, we develop multivariate PLS calibration models to compute multicomponent chiral compositions in unknown solutions. The validated models are applied to phase diagram determination in the R/S/S-MA/MeCN quaternary system represented as a tetrahedron plot in Figure 5. First, we detail the results regarding the spectral data and the calibration model specificities. Then, we present the revaluation of three solid–liquid ternary phase diagrams at 9 °C, represented on the faces of the tetrahedron involving the solvent. We start with the phase diagram between R and S enantiomers forming a racemic compound RS (Figure 5a), then between S and S-MA forming a 1:1 enantiospecific cocrystal S:S-MA (Figure 5b), and next between R and S-MA forming no cocrystal (Figure 5c). Finally, the inside of the tetrahedron (Figure 5d) is investigated in detail for the first time as our models allow quantification of quaternary compositions, with the view to understand the solid phase stabilities and their solubilities as a function of component compositions.

### 3.1 Multivariate PLS Calibration Model Development from UV-CD Spectra

#### 3.1.1 Spectral Data in the Quaternary System

**Figure 6:**
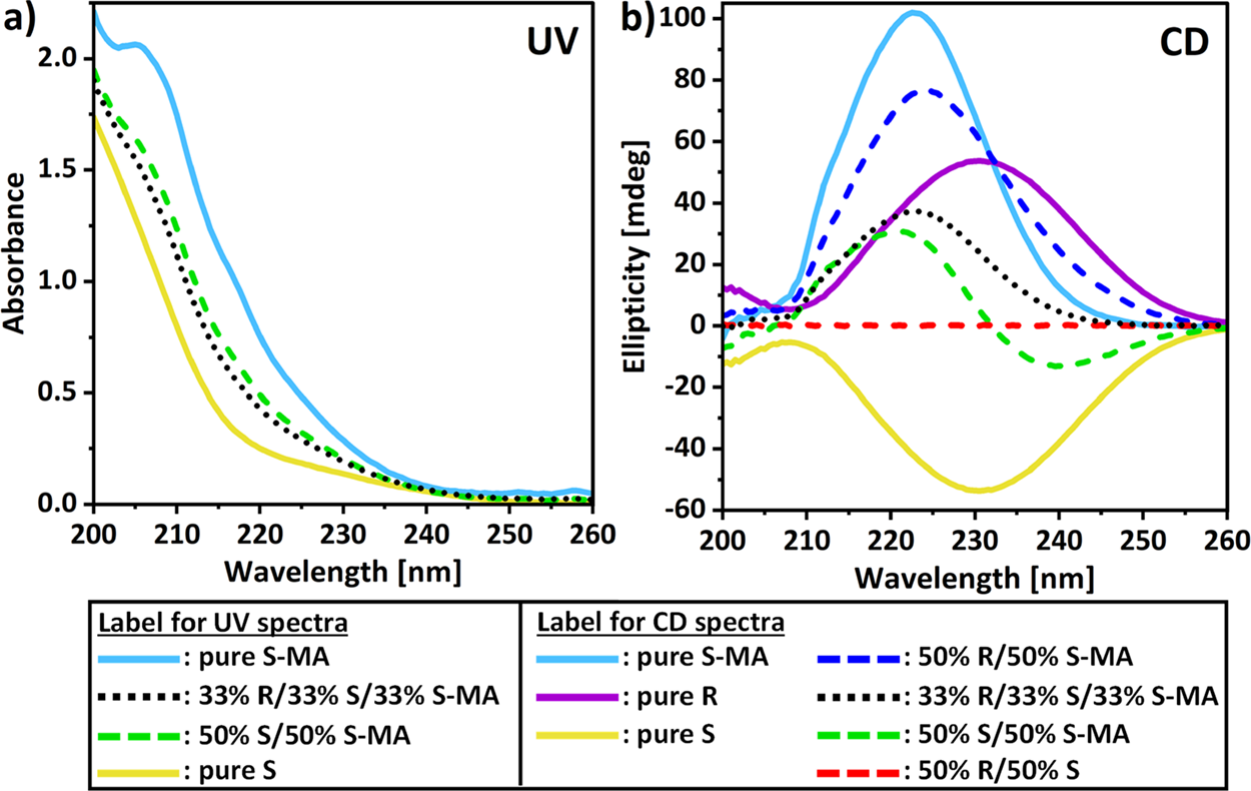
(a) UV and (b) CD spectra of solutions with a normalized total concentration of 4 mg/mL: pure components (solid lines), binary mixtures (dashed lines), and ternary mixtures (dotted lines).

To treat spectral data from UV and CD, defining a wavelength range where all dissolved molecules absorb UV is necessary. Since the solvent MeCN absorbs UV below 195 nm and R, S, and S-MA do not absorb above 260 nm, the optimal wavelength range is chosen to be from 200 to 260 nm. The whole region is used for composition prediction using multivariate methods. In Figure 6a, UV spectra for several calibration samples are represented. UV spectroscopy does not distinguish between the R and S enantiomers, and both molecules yield an absorption peak below the chosen wavelength range with a large part of the tail of this peak visible from 200 to 250 nm in Figure 6a (yellow solid line). S-MA shows similar UV absorption behavior but additionally has a shoulder at 205–216 nm (Figure 6a, light blue solid line). Because of the significant overlap in UV spectra of pure R/S and pure S-MA, the influence of each component in R/S and S-MA mixtures is difficult to distinguish but can be observed in the resulting spectral shape. Therefore, it assesses the necessity of using multivariate calibration for modeling, as it considers the effects of composition changes on the whole wavelength range at the same time. Both the normalized spectral shape for 50% S/50% S-MA (Figure 6a, green dashed line) and 33.3% of R, S, and S-MA (Figure 6a, black dotted line) mixtures highlight this influence. The more S-MA a sample contains, the more the inflections are marked in the resulting spectra.

Figure 6b shows the CD spectra of the same calibration samples, where we observe that CD distinguishes both enantiomers. R and S give positive and negative symmetrical responses with peak extrema at around 230 nm (purple and yellow solid lines). The 50%/50% mixture of R and S yields a flat line signifying the presence of the equal amount of both enantiomers (red dashed line). S-MA has a positive CD spectrum with a peak at 223 nm (Figure 6b, light blue solid line). Significant overlap can be seen between the mixtures of R, S, and S-MA, leading to different spectral shapes based on the component ratio. For instance, an equimolar ratio between R, S, and S-MA results in a CD spectrum of the same shape as pure S-MA with a peak at 223 nm but with the intensity being a third, for the same total concentration (black dotted line). Therefore, despite the overlap, the shapes and intensities of the CD spectra show information on both the concentration difference between R and S and the S-MA concentration.

#### 3.1.2 Multivariate PLS Calibration Model Specificities

The results of PLS calibration models for the quantitative prediction of *x*_S+R_, *x*_S–R_, and *x*_S-MA_ are summarized in Table 1. Their reliabilities and accuracies were evaluated internally and externally using cross-validation and the validation datasets. The root mean square error of prediction (RMSEP) is computed to estimate the error in predicting the measured values of a known sample, while the root mean square error of cross-validation (RMSECV) estimates the error in predicting the values of a calibration sample. The models are also tested by *R*^2^ (goodness of fit) and *Q*^2^ (goodness of prediction) values. *R*^2^ gives the amount of variance explained by the model, and *Q*^2^ gives the amount of variance predicted by the model. Both PLS models required only two LVs to compress the spectral data variables and capture the variance in the data, while giving good predictions with high linearity (*R*^2^ > 0.99, *Q*^2^ > 0.97). The high accuracy is highlighted by the levels of the RMSEP and RMSECV that show a lower order of magnitude for mass fraction errors than the mass fraction values of the calibration samples (see the Supplementary Information, Table S1).

**Table 1:** Results of PLS Calibration Models for UV and CD Spectral Data Acquired in the 200–260 nm Range Describing the Accuracy in the Composition Prediction^a^

data	method	value predicted	no of LVs	RMSEP (×10^–6^) (g/g)	RMSECV (×10^–6^) (g/g)	*R*^2^	*Q*^2^
UV	PLS	*x*_S+R_	2	16.3	13.0	0.997	0.977
		*x*_S-MA_		14.5	12.0		
CD	PLS	*x*_S–R_	2	12.0	1.16	0.998	0.986
		*x*_S-MA_		15.4	11.1		

a Results are the number of latent variables (LVs) required, the root mean square error of prediction (RMSEP), the root mean square error of cross-validation (RMSECV), the goodness of fit *R*^2^, and the goodness of prediction *Q*^2^.

**Figure 7:**
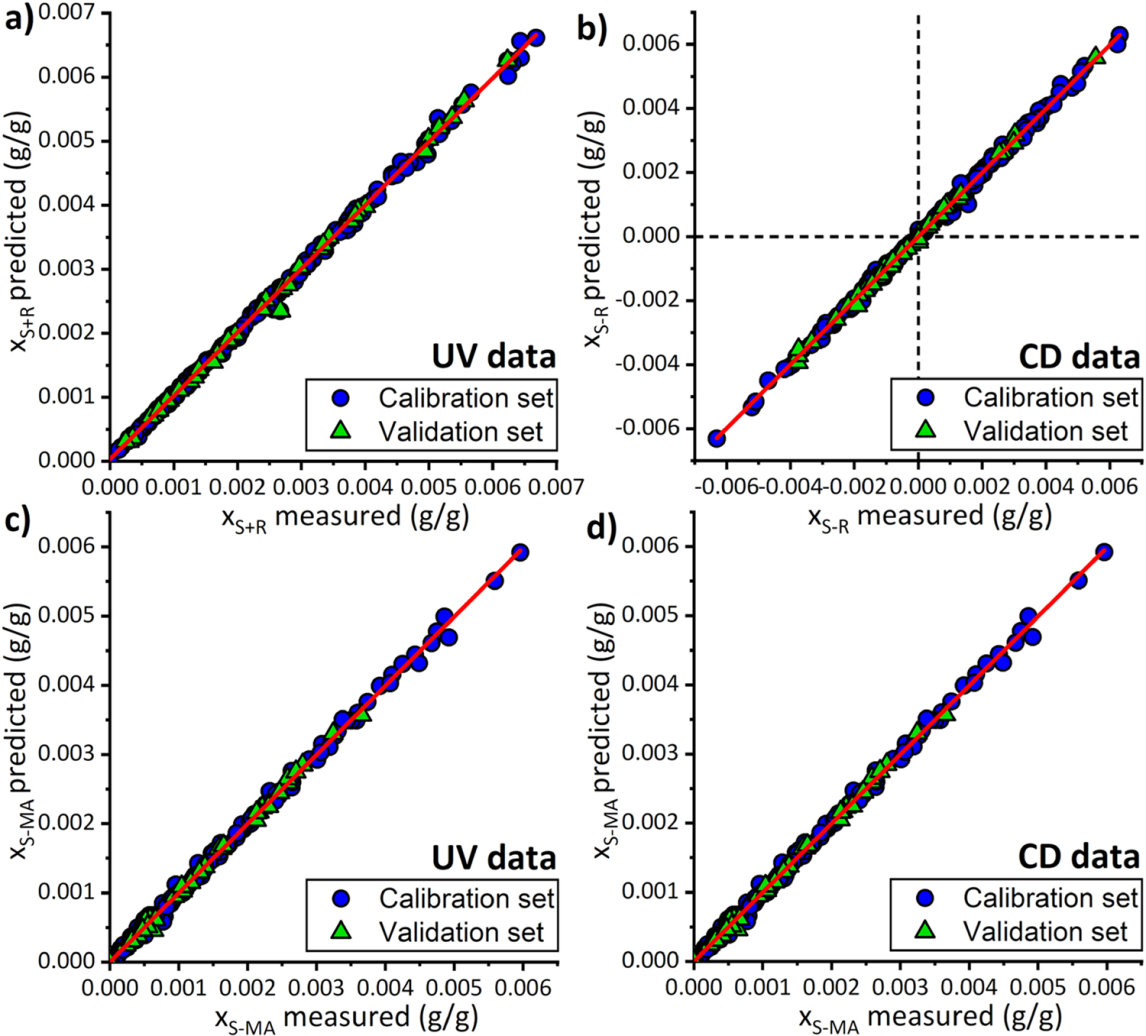
Plots for experimental values of calibration samples versus their predicted values through the calibration models for (a) Total mass fraction in enantiomers *x*_S+R_ (UV data). (b) Differential enantiomers mass fraction enantiomers *x*_S–R_ (CD data). (c) Mass fraction in S-MA *x*_S-MA_ (UV data). (d) Mass fraction in S-MA *x*_S-MA_ (CD data).

Figure 7 shows the predicted values of calibration samples through the calibration models versus their actual values for *x*_S+R_ (a), *x*_S–R_ (b), and *x*_S-MA_ (c, d), to visualize the goodness of fit. It can be observed that the split of samples between validation sets (green triangles) and calibration sets (blue points), performed using the Kennard–Stone algorithm,^72^ is uniform in the distribution and therefore representative. The values of *x*_S–R_ from CD in Figure 7b range from positive to negative, representing an excess of S and R in the sample, respectively. Very strong linearity along the diagonal lines in red can be seen in the plots for all samples, meaning that prediction is very close to the actual value. The linearity relates to the RMSEP and RMSECV values that quantify the error on how much samples from the calibration sets and validation sets deviate from the diagonal line, therefore giving an estimation of the average error in a prediction. There is no significant difference between *x*_S-MA_ predicted from both the UV and CD measurements (Figure 7c,d), with the RMSEP and RMSECV values being very similar, thus showing the accuracy and consistency of the models. However, the PLS model with CD data gives the best prediction with the lowest RMSECV and *R*^2^. Therefore, the *x*_S-MA_ value from CD is always used in calculations for accuracy.

The UV-CD model predictions are compared with results obtained from the gravimetric method for 28 compositions of different ratios in S and S-MA that were analyzed simultaneously by UV-CD spectroscopy. The percentage error  is used for comparison, with *X* being the total solubility obtained with the UV-CD model result, and *Y* the total solubility from the gravimetric method, on the same saturated solution. It shows a mean δ of 2.09% between the two methods on the total solubility, with a standard deviation of 1.47% (see the Supplementary Information, Table S2). Even though gravimetry is not an accurate quantification method, particularly when using a single measure, it relies on a physical measurement and therefore confirms that our multivariate calibration models do not have a bias in their calculations. These validated calibration models allow accurate determination of the mass fractions of unknown solutions in R (*x*_R_), S (*x*_S_), S-MA (*x*_S-MA_), and MeCN (*x*_MeCN_), and therefore, they are used for computing the phase diagram data.

### 3.2 Isothermal Ternary Phase Diagrams

#### 3.2.1 Ternary System of R/S/MeCN

**Figure 8:**
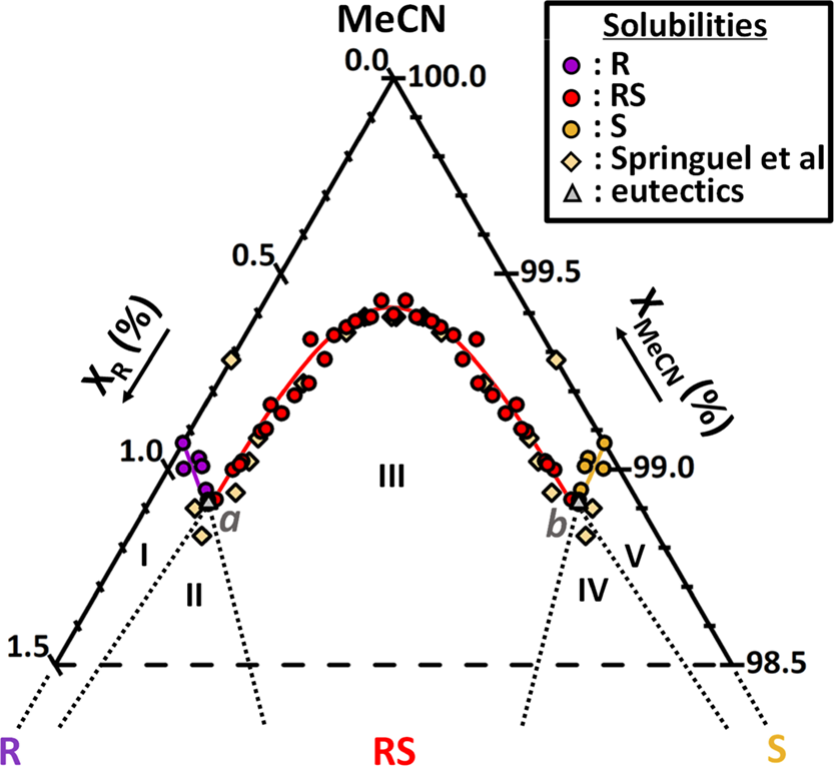
Isothermal ternary phase diagram of R and S in MeCN at 9 °C showing a racemic compound system. Regions I, III, and V are, respectively, the stability domains in which an overall composition phase splits into a saturated solution and, respectively, the solid R (purple solubility points), racemic solid RS (red solubility points), and the solid S (yellow solubility points). Regions II and IV are triphasic domains between the racemic compound RS, a solution of eutectic composition (gray triangle) and R and S, respectively. Above the solubility lines is the single-phase domain of the undersaturated solution. Dotted lines are boundaries between stability domains. Beige diamonds are the solubility points from the Springuel et al. study obtained with achiral and chiral chromatography.^35^ Note that the phase diagram is zoomed in to the solvent corner. Data points used for the construction of this diagram are detailed in the Supplementary Information (Table S4). Eutectic points *a* and *b* were measured experimentally with a composition presenting S and RS in stable suspension.

In the R/S/MeCN system, the stable solids consisting of pure R, pure S, and pure racemic compound RS are expected to crystallize at equilibrium. In total, 26 equilibrated solution compositions, with enantiomeric excess (*E*) values from 0 to 100%, are computed from experimental results. Due to symmetry along racemic compositions in enantiomeric systems, 26 additional points corresponding to negative values of enantiomeric excess (*E*) are deduced from the mirror projection of the first 26 points. The isothermal ternary phase diagram of R and S in MeCN at 9 °C is plotted in Figure 8. Solubility lines correspond to the typical shape of a stable racemic compound in an isothermal ternary system and solid phases in equilibrium are confirmed. This phase diagram is in excellent agreement with previous data obtained with a combination of achiral and chiral chromatography methods (Figure 8, beige diamonds).^35^

The eutectic points *a* and *b* (Figure 8, gray triangles) are obtained with an experimental composition presenting S and RS in stable suspension. These points fit perfectly with the intersection of neighboring solubility curves. Experimental solubility values of pure R, S, and RS solids, with compositions of eutectic points *a* and *b*, are compiled in the Supplementary Information, Table S3. All data point compositions with related solid phases identified at equilibrium used in Figure 8 are given in the Supplementary Information, Table S4. Only the pure enantiomer solubility differs slightly from previous data.^35^ However, we note that our value is confirmed through the repetition of four measurements in different saturated solutions of pure S, with UV-CD and the gravimetric method used to compare the model’s prediction. Both methods lead to the same value with about 0.5 mg/mL variation (see the Supplementary Information, Table S5).

No significant solubility modification effect is observed for pure R (purple) and pure S (yellow) solid solubility points due to the presence of the other component as *X*_R_ and *X*_S_ stay relatively constant. The solubility increase for *X*_R_ and *X*_S_ values at the eutectic points *a* and *b* is only 2%. Where the racemic compound RS equilibrates (red points), its solubility (*X*_R_ × *X*_S_)* shows an important curvature depicting lower *X*_R_ values than *X*_R_ at the eutectic *a*, down to a minimal total solubility (*X*_R_ + *X*_S_) = 0.6% for pure RS at 1:1 stoichiometry between R and S. The solubility of the pure enantiomer in the pure solution is 1.5 times higher than pure RS total solubility in a racemic solution. Maximum total solubilities are reached in eutectic points, where the total solubility is 1.2 times higher than pure R and S and 1.8 times higher than pure RS solubility.

#### 3.2.2 Ternary System between S/S-MA/MeCN

**Figure 9:**
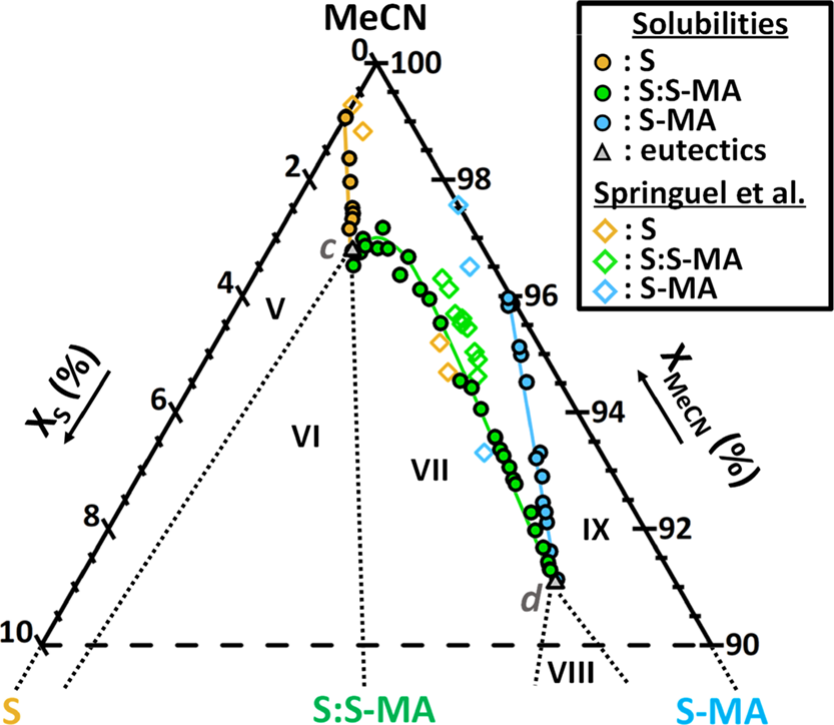
Isothermal ternary phase diagram of S and S-MA in MeCN at 9 °C showing an enantiospecific cocrystal system. Regions V, VII, and IX are the stability domains in which an overall composition phase splits into a saturated solution and the solid S (yellow solubility points), the cocrystal S:S-MA (green solubility points), and the solid S-MA (blue solubility points), respectively. Regions VI and VIII are triphasic domains between the cocrystal S:S-MA, a solution of eutectic composition (gray triangle) and S and S-MA, respectively. Above the solubility lines is the single-phase domain of the undersaturated solution. Dotted lines are boundaries between stability domains. Diamonds are the solubility points from Springuel et al. study obtained with achiral and chiral chromatography.^35^ Note that the phase diagram is zoomed in to the solvent corner. Data points used for the construction of this diagram are detailed in the Supplementary Information (Table S6). Eutectic point *c* was measured experimentally in a composition presenting S and S:S-MA in stable suspension. Eutectic point *d* was estimated at the intersection of converging solubility curves.

In the S/S-MA/MeCN system, the stable solids consisting of pure S, pure S-MA, and pure 1:1 enantiospecific cocrystal S:S-MA are expected to crystallize at equilibrium. Experimental solubilities are computed from experimental results of 55 equilibrated suspensions of varying ratios between S and S-MA in MeCN. The isothermal ternary phase diagram at 9 °C is plotted in Figure 9, zoomed in to the solvent corner. The phase diagram corresponds to a stable 1:1 cocrystal forming system between S and S-MA. As the theoretical line between the 1:1 stoichiometry of the S:S-MA solid phase and the pure solvent MeCN crosses the solubility curve of S/S-MA (green), the cocrystal exhibits a congruent solubility at 9 °C, meaning that it forms a stable suspension in solutions with the same stoichiometry as the cocrystal.

The eutectic point *c* is obtained at an experimental composition presenting S and S:S-MA in stable suspension. It fits well with the intersection of neighboring solubility curves. The eutectic point *d* is estimated at the intersection of converging solubility curves. In Figure 9, the phase diagram solubility points and domain shapes differ slightly from previous data and their interpretation with fewer data points on the same system by Springuel et al.,^35^ as they suggested the cocrystal to have an incongruent solubility (diamond points). Here, with more data points presented, and an experiment resulting in eutectic solution composition *c* with S and S-MA solids in suspension, we revaluated the stability domains. A shift can also be observed between some of their solubility data and ours, even in pure component solubilities. We checked the latter through the repetition of four measurements in different saturated solutions of pure S and pure S-MA with the UV-CD model and the gravimetric method that was used when validating the model’s predictions by comparison with an external method. It gives consistent values and negligible variations (see the Supplementary Information, Table S5). Moreover, 28 saturated solutions from our ternary system were validated simultaneously by the gravimetric method (see the Supplementary Information, Table S2). Therefore, we propose an accurate revaluation of the phase diagram using consistent results. Experimental solubility values of pure S, S-MA, and S:S-MA solids, with compositions of eutectic points *c* and *d*, are compiled in the Supplementary Information, Table S3. All data point compositions with related solid phases identified at equilibrium used in Figure 9 are given in the Supplementary Information, Table S6.

A strong effect on the solubility of pure S solid is observed (yellow) as a function of the concentration of S-MA: the solubility *X*_S_ at the eutectic point *c* is 2.1 times higher than that in the pure solvent. The total solubility at eutectic point *c*, including the S-MA concentration, is 3.5 times higher than that in the pure solvent. Similarly, pure S-MA solid solubility points (blue) are increased by the presence of S, up to a solubility *X*_S-MA_ at eutectic point *d* that is 1.8 times higher than for pure S-MA solubility, while the total solubility is 2.2 times higher than for S-MA in the pure solvent. The solubility (*X*_S_ × *X*_S-MA_)* of the S:S-MA cocrystal (green points) decreases as a function of concentration of S and S-MA, from a maximum value at the eutectics, down to a minimum solubility point that is the pure S:S-MA congruent solubility value at 1:1 stoichiometry between S and S-MA, for a minimal total solubility (*X*_S_ + *X*_S-MA_) = 3.2%. The solubility of S-MA in pure solvent is 1.3 times higher than the total solubility of S:S-MA, whose *X*_S-MA_ is divided by 2.5 compared to the pure S-MA solubility. However, the total solubility of pure S:S-MA is 3.4 times higher than pure S, with *X*_S_ being 1.7 times the pure S solubility. The possible explanations for the increase in solubility of pure S and pure S-MA solids, with the presence of the other component in solution, are most likely due to favorable intermolecular interactions between components in solution. Nevertheless, solution complexation is also a possible reason as it has been reported to occur for some cocrystal components.^74^

#### 3.2.3 Ternary System between R/S-MA/MeCN

**Figure 10:**
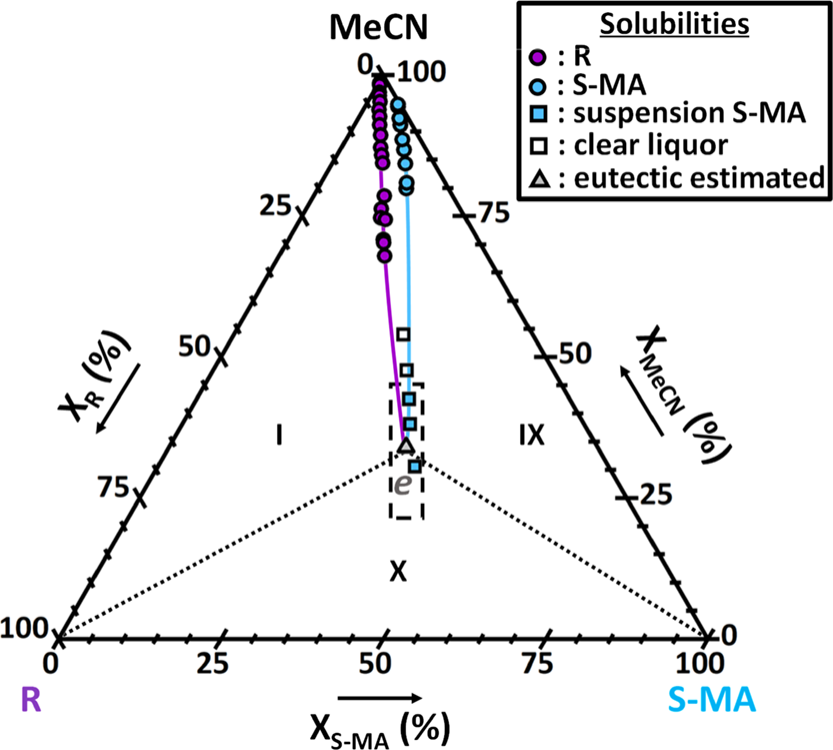
Isothermal ternary phase diagram of R and S-MA in MeCN at 9 °C showing a single eutectic equilibrium. Regions I and IX are the stability domains in which an overall composition phase splits into a saturated solution and the solids R (purple solubility points) and S-MA (blue solubility points), respectively. Region X is the triphasic domain between R, S-MA, and a solution of eutectic composition *e* (gray triangle). Above the solubility lines is the single-phase domain of the undersaturated solution. Dotted lines are boundaries between stability domains. Blue squares correspond to overall compositions of which, due to the high viscosity, only the equilibrated solid could be sampled for XRPD analysis to be identified as S-MA. White squares correspond to sample compositions in which no solid was present after the equilibration period. The dashed box is the region in which eutectic point *e* is estimated, from the extrapolation of solubility curves and suspensions obtained at blue squares. The center of the box is chosen as the most likely estimation. Data points used for the construction of this diagram are detailed in the Supplementary Information (Table S7).

In the R/S-MA/MeCN system, the stable solids consisting of pure R and pure S-MA are expected to crystallize at equilibrium. Experimental solubilities are computed from experimental results of 28 equilibrated suspensions of varying ratios between R and S-MA in MeCN. The isothermal ternary phase diagram at 9 °C is plotted in Figure 10, zoomed in to the solvent corner. Contrary to the S/S-MA/MeCN system, no cocrystal forms between R and S-MA as the solubility lines seem to converge to a single eutectic point *e* and no new solid phase is identified in the experiments. Therefore, it confirms the enantiospecific nature of the S:S-MA cocrystal identified from the Springuel et al. study.^31^

Solubility lines show a strong influence of the components on each other’s solubility, with the total solubility increasing sharply in mixtures. The solubility of R is increased more by the concentration of S-MA than the solubility of S in the S/S-MA/MeCN system. This strong increase of the solubility of R with the S-MA concentration, coupled with the absence of cocrystal formation, is causing eutectic point *e* to be a deep eutectic. This strong affinity between components was already reported in the binary system of R and S-MA,^31^ whose binary eutectic temperature of around 32 °C is about 100 °C deeper than the pure R and pure S-MA melting points. Therefore, in the R/S-MA/MeCN ternary system at 9 °C, it induces a small triphasic domain between R, S-MA, and a saturated liquid of eutectic composition *e* that is at a very high equilibrium concentration. This leads experimentally to a big increase in sample viscosity as solubility increases strongly for compositions close to the eutectic point *e*, making it difficult to estimate as the solutions are too viscous to be accurately sampled for liquid analysis. Trial experiments to screen eutectic point *e* are represented in Figure 10 by square points, which correspond to five highly concentrated suspensions left at 9 °C for more than 3 weeks, after complete dissolution and seeding with a small amount of R and S-MA solids. For three compositions (blue squares), a very small amount of solid phase crystallizes in the highly viscous liquids. The isolated solid, characterized using XRPD, is pure S-MA despite a low intensity signal because of the small amount of solid recovered. For the two other compositions (white squares), the liquor remains clear with no crystallization happening, it is then assumed they belong to the undersaturated solution domain. These results help to estimate roughly the extension of solubility lines and to define a compositional region in which eutectic point *e* is positioned. For the system representation and description purposes, the composition of eutectic point *e* is an approximation. Experimental solubility values of pure R, S-MA, and estimation of eutectic point *e* are compiled in the Supplementary Information, Table S3. All data point compositions with related solid phases identified at equilibrium used in Figure 10 are given in the Supplementary Information, Table S7.

An important solubility increase effect is observed for the pure R solid solubility points (purple) as *X*_R_ values increase due to the increasing presence of S-MA, up to an estimated value of about 32 times higher than pure R solubility at the estimated eutectic point *e*. The total solubility at eutectic point *e* is about 70.7 times higher than for pure R in MeCN. Similarly, pure S-MA solid solubility points (blue) are increased by the presence of R, up to an *X*_S-MA_ value being about 9 times higher than pure S-MA solubility at eutectic point *e*, whose total solubility is about 16 times higher than for pure S-MA in MeCN. The solubility behavior of the R/S-MA/MeCN system is therefore very different from that of the S/S-MA/MeCN system, with a stronger impact of R solubility with S-MA concentration than it is for S solubility, and no cocrystal forming. This difference will cause a huge asymmetry in the quaternary system. Favorable intermolecular interactions between components in solution could be the reasons why the solubility of pure R and pure S-MA solids increase with the presence of the other component in solution. Another possibility is the occurrence of solution complexation between the components.^74^

### 3.3 Quaternary System with R/S/S-MA in MeCN at 9 °C

After investigating the three isothermal ternary phase diagrams that correspond to each face of the quaternary tetrahedron, the full isothermal quaternary phase diagram has been explored using 168 equilibrated quaternary suspensions distributed inside the tetrahedron. In this system, all stable solids from the ternary systems, consisting of pure R, S, S-MA, RS, and S:S-MA are expected to crystallize at equilibrium. As for every phase diagram, quaternary phase diagrams follow the Gibbs phase rule,^75^ which defines the number of degrees of freedom, *v*, that are independent intensive parameters required to define an equilibrium state. The Gibbs phase rule is expressed as
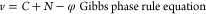
where *C* is the number of independent components (in this case *C* = 4), *N* is the number of intensive parameters that the system depends on (in this case *N* = 0), and φ is the number of phases in equilibrium, giving *v* = 4 – φ for this system.

**Figure 11:**
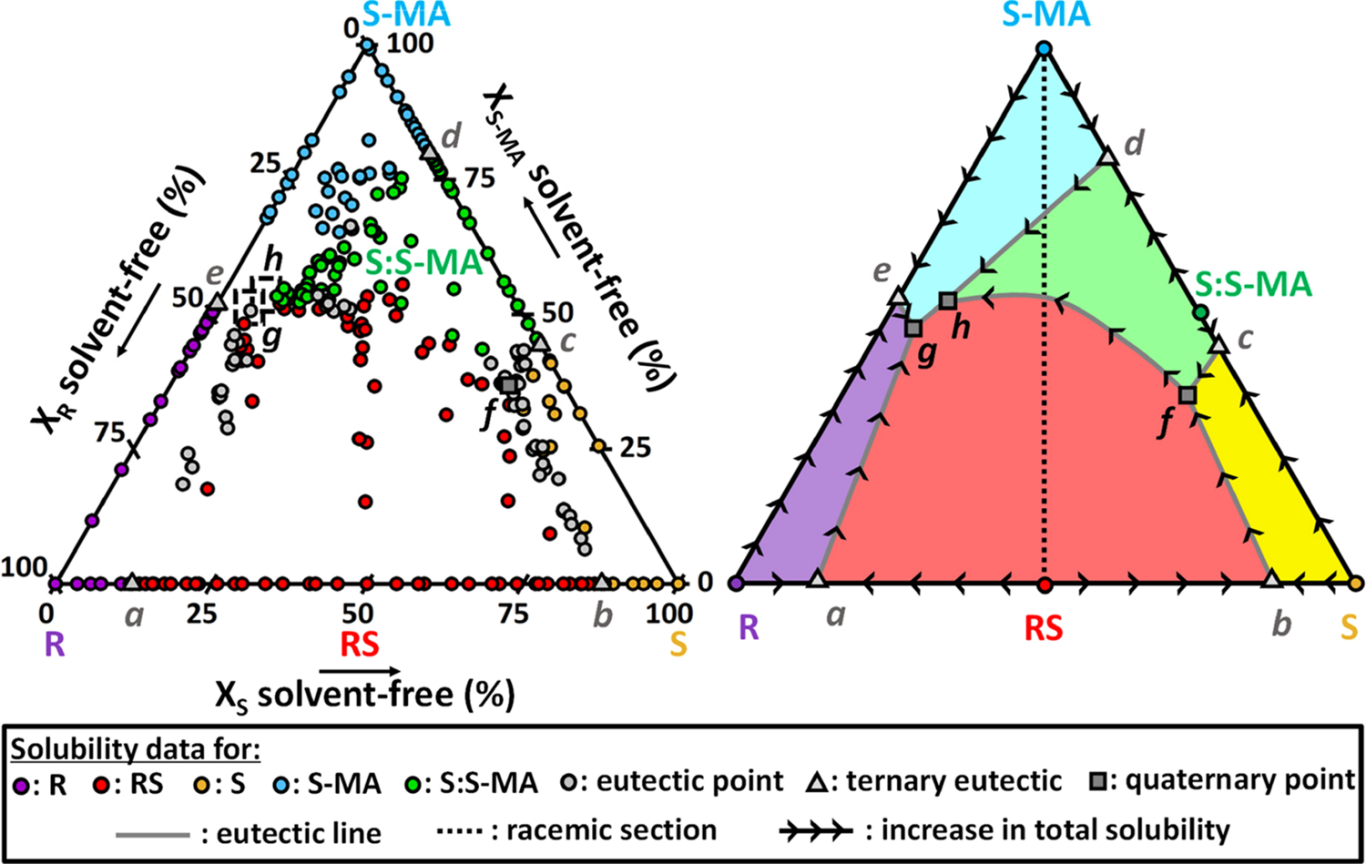
Left: projection of experimental results from the equilibration experiments showing the solvent-free solution compositions in the quaternary phase diagram R/S/S-MA/MeCN at 9 °C. The colors of the points indicate the solids that are equilibrated with a saturated solution. Dashed boxes are compositional zones in which quaternary points are not measured but expected. All data used in the quaternary phase diagram can be found in the Supplementary Information (Table S8) Right: interpretation of results projection into solubility surfaces, eutectic lines, and quaternary points. Arrows point toward the direction of increasing total solubility. The dotted black line represents the racemic section in the quaternary (equimolar ratio between R and S).

The maximum total solubility point in this quaternary phase diagram is measured to be about 140 times higher than the minimal total solubility point, making it impossible to clearly represent the full characteristics of the quaternary in the 3D phase diagram. Therefore, a solvent-free projection of solubility surfaces is used in Figure 11 (left) to display all experimental points from the quaternary system and related ternary systems. By removing the dependency on the solvent concentration, solubility data can be shown in a two-dimensional plot where points are positioned based on their relative solvent-free molar ratio in dissolved components (R, S, and S-MA). Explanations about how solvent-free projections are performed from phase diagram solubility points are provided in the Supplementary Information (Figure S3). The points in Figure 11 (left) are colored according to the solid phase(s) identified in equilibrium for each saturated solution. The points identified as belonging to biphasic domains (*v* = 2) correspond to a split of an overall composition between a saturated solution and one of the solids R (purple), S (yellow), S-MA (blue), RS (red), or S:S-MA (green). When two solids are identified at equilibrium (light gray), the points belong to a triphasic domain (*v* = 1) of which the measured saturated solution is a eutectic composition, similarly to previously measured eutectics in ternary sections (light gray triangles). A maximum of three solids can be identified as stable in a suspension at equilibrium (dark gray squares), that is therefore part of a quadriphasic domain (*v* = 0) of which the measured saturated solution is the unique possible liquid composition, referred here as a quaternary point.

Figure 11 (right) is our interpretation of experimental points in the solvent-free projection. Biphasic domain points cover a region defining a solubility surface, whose color is chosen depending on the related stable solid. These regions have boundaries that are a part of the figure sides corresponding to the solid solubilities in the ternary phase diagrams (black lines) down to a ternary eutectic point (light gray triangle). For example, the solubility surface of pure S (yellow) presents the solubility data from R/S/MeCN and S/S-MA/MeCN ternaries, from pure S solubility to ternary eutectic points *b* and *c*. The boundaries between regions are also eutectic lines (dark gray) that link eutectic compositions associated to triphasic domains that equilibrate the two same solids, each being from the neighboring solubility surfaces. The eutectic lines can link a ternary eutectic point with a quaternary point that presents the two same solids at equilibrium. For instance, between ternary eutectic *b* showing S and RS solids equilibrating in the liquid, and quaternary point *f* equilibrating S, RS, and S:S-MA in suspension. It can also link two quaternary points presenting the same two solids in their equilibrated suspensions, such as quaternary points *f* and *h* both equilibrating RS and S:S-MA among their stable solids. Quaternary points always correspond to the intersection of three eutectic lines, as they represent the solution of unique composition possible in a quadriphasic domain (*v* = 0), saturated in the three stable solids in suspension, according to the Gibbs phase rule.^75^ For example, the quaternary point *f* is the saturated solution corresponding to RS, S, and S:S-MA in stable suspension. It is identified experimentally with an XRPD result presenting the three solids signatures. We can observe it fits perfectly with the convergence of three eutectic lines equilibrating two of these solids.

The arrows shown in Figure 11 (right) are pointing toward the direction of increasing total solubility, to represent the relative quantity of solvent in the saturated solutions based on experiments results. The pure solid phases are always presenting a total solubility lower than the ternary eutectic points they are linked to, therefore with an arrow pointing down to them. The ternary eutectic points themselves have a lower total solubility than the quaternary point they are linked to and, consequently, an arrow directed toward them. For instance, the total solubility of quaternary point *f* is 3.7 times higher than ternary eutectic point *b* and 1.3 times higher than ternary eutectic point *c*. Its solubility in S is the highest of the whole stability domain of S, being 2.3 times higher than pure S solubility. Between, two quaternary points linked, there is no rule regarding the direction of evolution of total solubility. Overall, S/S-MA/MeCN ternary system (S to S-MA axis) exhibits a much lower solubility than the R/S-MA/MeCN one (R to S-MA axis). Figure 11 (right) reflects this huge difference by a substantial asymmetry in the quaternary system. All solubility surfaces dive toward compositions close to the estimated eutectic point *e*, as shown in the direction of the eutectic lines. The lack of experimental data in Figure 11 close to eutectic point *e* is again due to viscous solutions, difficult to equilibrate and sample. Four eutectic lines are converging in this region but the way they meet cannot be determined precisely. However, because of the Gibbs phase rule,^75^ it is impossible for four phases to be in equilibrium with one composition in such an isothermal isobaric quaternary system. Therefore, there must exist the two quaternary points, *g* and *h*, each being the intersection of three eutectic lines. The compositional zones in which they are expected can be estimated from the extension of the eutectic lines, as represented in Figure 11, to compute an approximate solvent-free ratio (see the Supplementary Information, Table S3). We also know that both total solubilities at *g* and *h* are higher than at eutectic point *e*, which we estimate to be approximately 6 g/mL MeCN. However, it is not possible to know whether *g* or *h* has the highest overall solubility, and therefore, the direction of the eutectic line in-between is unknown. Experimental solubility values of all pure solid phases, ternary eutectic points, and quaternary points are compiled in the Supplementary Information (Table S3). Compositions of all saturated solution points in the quaternary phase diagram can be found in the Supplementary Information (Table S8).

**Figure 12:**
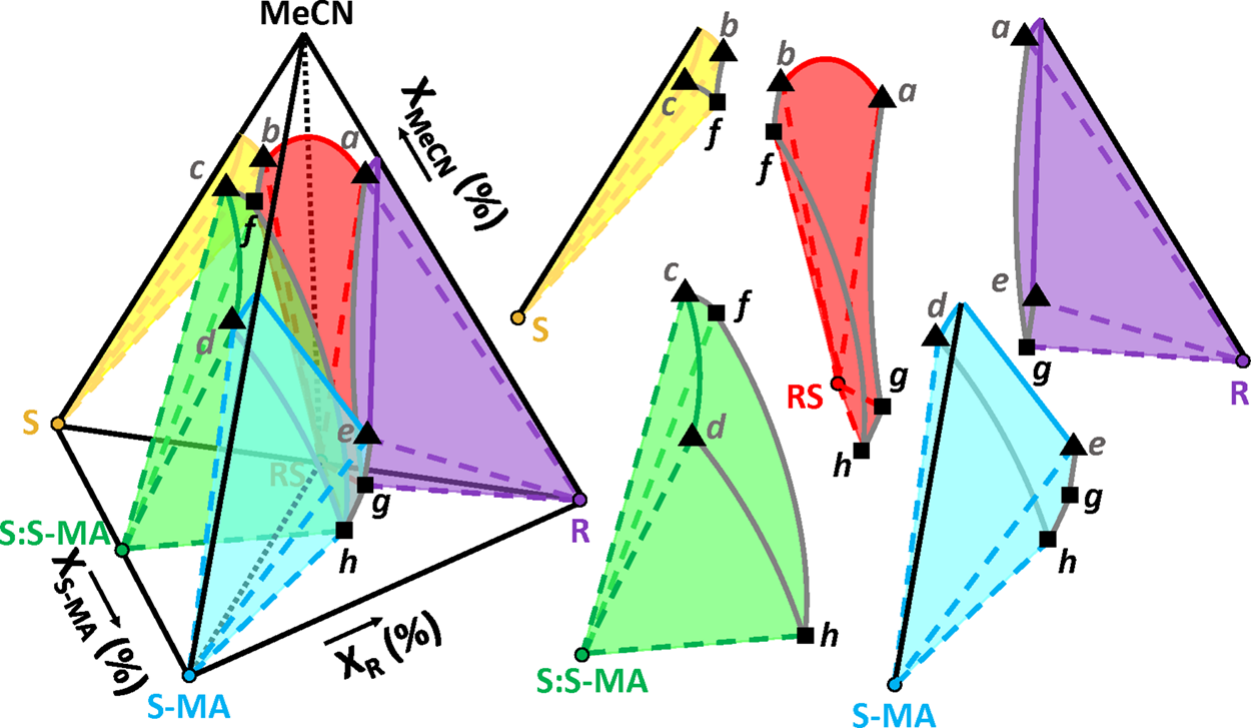
Graphical interpretation, not to scale, of the R/S/S-MA/MeCN quaternary phase diagram and the expanded view of biphasic stability domains of pure solid phases with their related colored solubility surface in equilibrium. Black triangles correspond to eutectic points in the ternary systems, and gray lines to eutectic lines originating from them, representing the equilibrium liquid composition lines saturated in two solid phases from adjacent domains. At the intersection of three eutectic lines are quaternary points (black squares) corresponding to the liquid composition saturated in the three neighboring solid phase domains. For clarity, the figure does not highlight triphasic domains (domain of tie-triangles linking eutectic lines to the two solids they are saturated in) and quadriphasic domains (domain whose boundaries are quaternary points linked with their three solids in equilibrium). The black dotted line indicates a cross-section of racemic composition (composition equal in R and S) in the quaternary.

Figure 12 shows a schematic interpretation of the full quaternary phase diagram as a tetrahedron plot, based on experimental data points plotted in the Supplementary Information (Figures S4 and S5) for different scales and viewing angles in the tetrahedron. Figure 12 is therefore not a representation to scale because of the large variation in total solubility in the full tetrahedron. We can identify the shapes and boundaries of the five biphasic stability domains, highlighting every possible composition that leads to the stable suspension of a pure stable solid (R, S, RS, S-MA, and S:S-MA) in a saturated solution through tie-lines. All possible saturated solutions spread as a solubility surface at the separation with the undersaturated solution domain whose apex is pure MeCN. Eutectic lines are identified on the intercept of two solubility surfaces and define a line of saturated liquids in both neighboring solid phases stability domains. The triphasic domains, not highlighted here for clarity, correspond to the zone of existence of suspensions following this equilibrium, linking saturated liquids from the eutectic lines to the two pure solids through tie-triangles. At the intersection of three eutectic lines are the quaternary points of unique liquid composition possible for suspension of three solids. The quadriphasic domain, not highlighted here for clarity, is a tetrahedron zone whose apexes are the three pure solids and the quaternary point, defining the existence zone of the suspensions. Inside, the phase compositions are not changing, only the mass balance between them is varying.

## 4 Discussion

In pure racemic compound systems, such as the ternary system R/S/MeCN, it is impossible to perform crystallization-enhanced chiral separation under stable conditions by starting from a racemic solution. Therefore, crystallization-enhanced chiral resolutions are performed using kinetic processes like preferential crystallization. As stable racemic compound systems occur in 90–95% of cases for crystallization equilibria of chiral molecules, it makes chiral resolution complex. Nonetheless, the symmetry in enantiomeric systems can be broken when adding a chiral component, such as S-MA, which can form enantiospecific solids, such as the S:S-MA cocrystal, even in racemic solutions. By determining the R/S/S-MA/MeCN quaternary phase diagram, we show the boundaries and shapes of the stability domains of all stable solids in the system. This leads to the understanding of the relation between overall composition and solid formation. We observe a huge asymmetry in the S/S-MA/MeCN ternary system, forming a stable S:S-MA cocrystal of low solubility, and the R/S-MA/MeCN ternary system highlighting a strong affinity between components in solutions, therefore reaching very highly concentrated solubility points. The consequence for the quaternary system is that the stability domain of S:S-MA is strongly skewed toward the opposite face of the tetrahedron, and therefore extends beyond the racemic composition. Indeed, in both Figures 11 and 12, we can observe that the racemic composition (Figure 11, dotted line) crosses the solubility domains of RS (red), S:S-MA (green), and S-MA (blue). This asymmetry highlights a zone along the racemic cross-section RS/S-MA/MeCN where the S:S-MA cocrystal is accessible for crystallization. A chiral resolution experiment in this zone has the advantage of being in stable conditions as the phase diagram describes thermodynamic equilibrium, with S:S-MA being the only solid present at equilibrium. This was experimentally proved by Springuel et al.^31^ To optimize chiral resolution in this zone, the knowledge of the entire quaternary phase diagram is required to define accurately the best working compositions. Based on the quaternary phase diagram data acquired here, it is possible to design process conditions during which the racemic compound RS and the chiral coformer S-MA as input can lead to obtaining only S:S-MA chiral cocrystal as output. Afterward, the cocrystal can be separated into its pure components and thereby the pure levetiracetam API (S), a nootropic drug used as an anticonvulsant to treat epilepsy. Therefore, the knowledge of complex phase diagrams can help in designing alternative chiral separation routes with crystallization for industry.

The need for complex chiral phase diagrams is limited due to the difficulty in quantifying chiral molecules in multicomponent chiral systems. With UV-CD spectroscopy and multivariate calibration models, we have managed to quantify different chiral molecules in solution with great accuracy and are not limited by the increasing number of chiral components. This enlarges the range of methods available for chiral molecule quantification, used here for phase diagram determination, and especially on multicomponent systems such as quaternary systems that were difficult to access until now. The UV-CD spectroscopy method can be extended to even more complex systems, if necessary, with appropriate multivariate calibration models. As multivariate techniques consider the variations in the whole spectrum and not at specific wavelengths, it is possible to take into account accurately the existing interactions in solution. For instance, the occurrence of complexation in solution can induce shifts in the spectra or potential changes in the molar absorptivity coefficient, which can be integrated in the multivariate calibration model. The UV-CD spectroscopy method could also be used for online monitoring of the solution composition during a crystallization process through in situ measurements or solution sampling of the liquid phase concentration and enantiomeric excess. The advantages of the UV-CD method are the absorbance detection of both chiral and achiral molecules, unaffected by the sample temperature, facile method development, and quick analysis. The sample preparation is minimal, requiring only sampling and dilution, and guarantees no possible physical/chemical degradation as it can be the case for other methods like chiral HPLC that introduces new solvents in contact with the sampled analytes. The same multivariate calibration models are needed for quantification of several components, and we prove the high consistency of data obtained through the present study. The limitations of the UV-CD method are the need for the molecules to absorb in the UV region, preferably in a region different from the solvent used. However, these criteria are already a requirement for chiral HPLC methods that use UV spectroscopy in their detectors. UV-CD cannot be applied to UV-sensitive molecules that become modified or degrade under UV light.^76−78^ Other chiroptical techniques like vibrational circular dichroism (VCD) or Raman optical activity can be a good alternative to UV-CD,^59^ as they present more pronounced spectra that arise from the vibration modes of the bonds, and thus are not limited by chemical degradation and absorption requirements. Both techniques also produce spectra, and therefore offer big possibilities in terms of data analysis with multivariate analysis to build quantification methods for chiral molecules.

## 5 Conclusions

A new multicomponent chiral quantification method using UV-CD spectroscopy and PLS calibration models was created to measure unknown compositions in up to three different chiral components in solution, with two being enantiomers. This method was used to design calibration models covering the R/S/S-MA/MeCN quaternary system. Three accurate ternary phase diagrams were measured, revising previous literature data. Moreover, with the newly possible quaternary composition quantification, the full quaternary phase diagram tetrahedron at 9 °C was proposed for the first time. It shows the equilibria of the two enantiomers forming a racemic compound RS and the enantiomer S forming an enantiospecific cocrystal S:S-MA with the chiral coformer S-MA. The calibration results show very high accuracy for models in predicting known compositions. They can predict the total mass fraction in enantiomers *x*_S+R_ with an RMSEP of 16.3 × 10^–6^ g/g, the differential mass fraction between enantiomers *x*_S–R_ with an RMSEP of 12.0 × 10^–6^ g/g, and the mass fraction in S-MA *x*_S-MA_ with an RMSEP of 15.4 × 10^–6^ g/g. The obtained phase diagram experimental results prove to be in good agreement with those obtained with other analytical methods such as HPLC and gravimetric analysis. The CD spectroscopy method is promising as it can be extended to wavelengths different from UV to build similar quantification models. Moreover, a higher number of different chiral molecules could be quantified in solution, with the appropriate multivariate calibration models on spectral data. Most chiral pharmaceutical compounds absorb in UV without degrading, and their concentration tends to have an influence on the spectrum, which is detectable by the PLS method in sufficient accuracy. Therefore, the method is potentially applicable to a large range of organic molecules. The accurate description of the quaternary phase diagram underlines a large asymmetry along the racemic composition, which shows the feasibility of a chiral separation process with enantioselective cocrystallization of levetiracetam under stable conditions. This highlights the necessity of complex multicomponent chiral phase diagram determination with precise methods, such as UV-CD spectroscopy and multivariate analysis.

## Supporting Information Available

Materials, models development data and phase diagrams data (PDF)

## Notes

All data underpinning this publication are openly available from the University of Strathclyde KnowledgeBase at: https://doi.org/10.15129/414d46ee-fe46-4ec0-9cbb-67f29c5efdf6
