# Supplementary material for: Multicomponent Chiral Quantification with Ultraviolet Circular Dichroism Spectroscopy: Ternary and Quaternary Phase Diagrams of Levetiracetam
Source: Mol Pharm. 2022 Dec 5;20(1):616–29. doi: 10.1021/acs.molpharmaceut.2c00825 (PMC9811458; doi:10.1021/acs.molpharmaceut.2c00825)
Supplement: Supplementary file 1 — mp2c00825_si_001.pdf [file mp2c00825_si_001.pdf]

Supporting Information:
"Multicomponent chiral quantification with UV circular dichroism spectroscopy: ternary and quaternary phase diagrams of Levetiracetam"

Maxime D. Charpentier[1], Raghunath Venkatramanan[1], Céline Rougeot[2], Tom Leyssens[3], Karen Johnston[4], Joop H. ter Horst[1,5]

1. EPSRC Centre for Innovative Manufacturing in Continuous Manufacturing and Crystallization (CMAC), University of Strathclyde, Technology and Innovation Centre, 99 George Street, Glasgow G1 1RD, U.K.

2. UCB Pharma SA, chemin du Foriest, 1420 Braine-L'Alleud, Belgium

3. Institute of Condensed Matter and Nanosciences, UCLouvain, Place L. Pasteur 1, Belgium.

4. Department of Chemical and Process Engineering, University of Strathclyde, James Weir Building, 75 Montrose Street, Glasgow G1 1XJ, U.K.

5. Univ Rouen Normandie, Laboratoire Sciences et Méthodes Séparatives (SMS), UR 3233, F-76000 Rouen, France

Table of contents

S1 – Materials 2
S2 – Model development data 3
S3 – Phase diagrams data 11

S1 – Materials

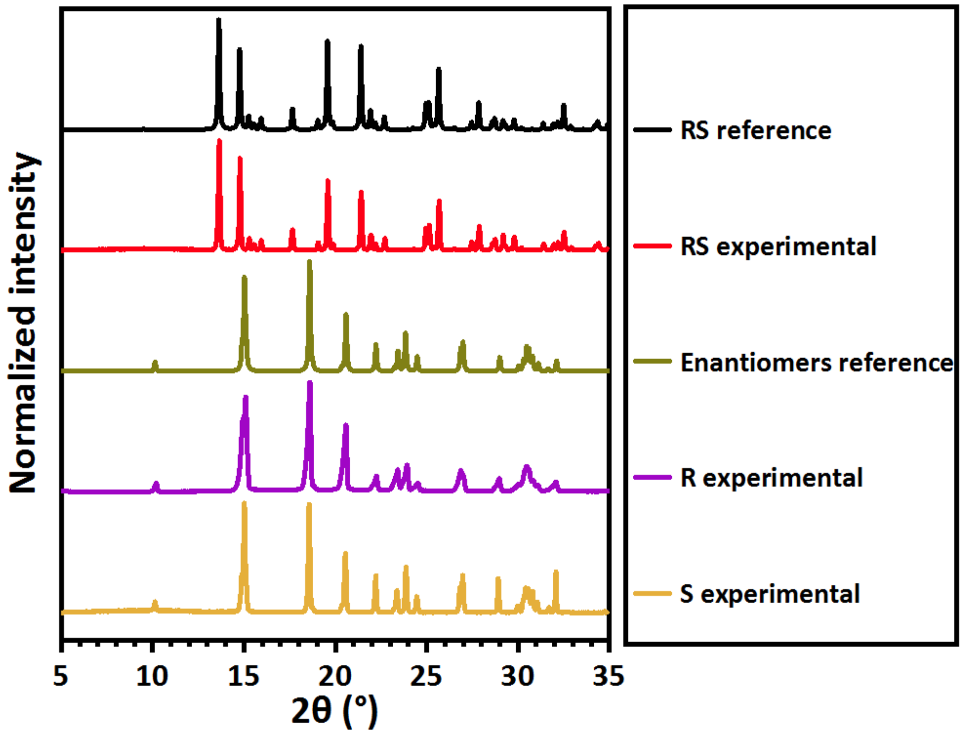

Figure S1: Experimental and reference XRPD patterns for solids of both enantiomers, and racemic compound RS.

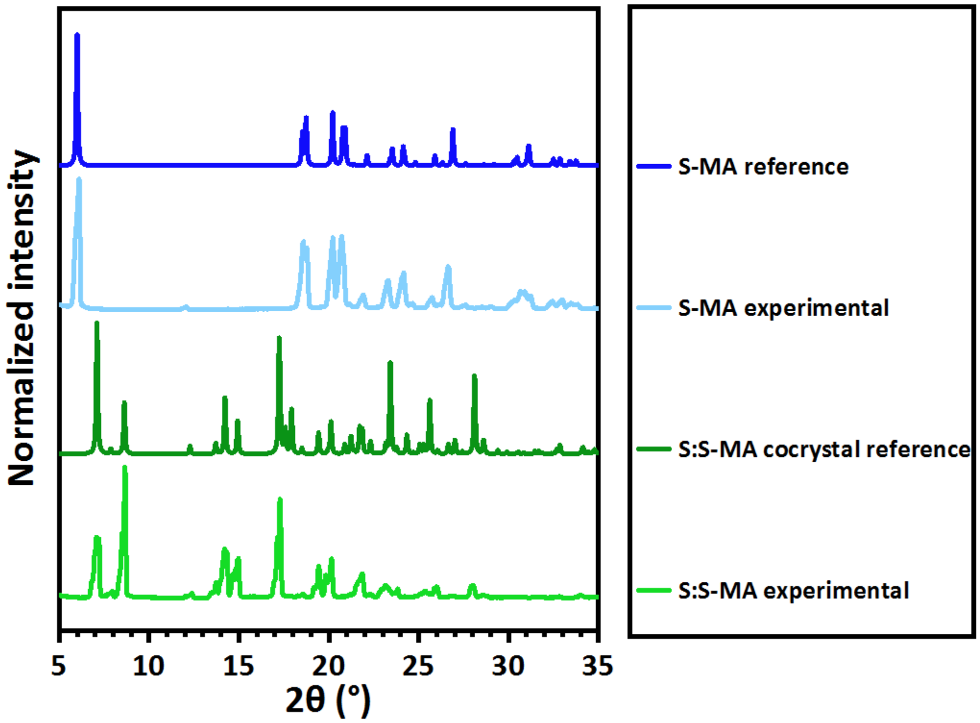

Figure S2: Experimental and reference XRPD patterns for solids of S:S-MA cocrystal and S-MA.

S2 – Multivariate calibration model development data

S2.1 – Mass fractions of calibration samples used

Table S1: Components mass fractions of calibration samples prepared in MeCN used for the calibration model.

Section	x_R	x_S	$x_{S\text{-}MA}$
Ternary R/S/MeCN	0	0.001266	0
	0	0.002657	0
	0	0.003947	0
	0	0.005210	0
	0	0.006307	0
	0.000115	0.001126	0
	0.000226	0.002213	0
	0.000348	0.003403	0
	0.000464	0.004538	0
	0.000580	0.005668	0
	0.000243	0.000990	0
	0.000492	0.001999	0
	0.000740	0.003008	0
	0.000985	0.004007	0
	0.001230	0.005004	0
	0.000381	0.000897	0
	0.000764	0.001801	0
	0.001150	0.002710	0
	0.001533	0.003612	0
	0.001915	0.004513	0
	0.000518	0.000813	0
	0.001038	0.001629	0
	0.001557	0.002444	0
	0.002080	0.003265	0
	0.002598	0.004078	0
	0.000636	0.000636	0
	0.001286	0.001286	0
	0.001931	0.001931	0
	0.002578	0.002578	0
	0.003219	0.003219	0
	0.001266	0	0
	0.002657	0	0
	0.003947	0	0
	0.005210	0	0
	0.006307	0	0
	0.001126	0.000115	0
	0.002213	0.000226	0

	0.003403	0.000348	0
	0.004538	0.000464	0
	0.005668	0.000580	0
	0.000990	0.000243	0
	0.001999	0.000492	0
	0.003008	0.000740	0
	0.004007	0.000985	0
	0.005004	0.001230	0
	0.000897	0.000381	0
	0.001801	0.000764	0
	0.002710	0.001150	0
	0.003612	0.001533	0
	0.004513	0.001915	0
	0.000813	0.000518	0
	0.001629	0.001038	0
	0.002444	0.001557	0
	0.003265	0.002080	0
	0.004078	0.002598	0
**Ternary S/SMA/MeCN**	0	0	0.001197
	0	0	0.002379
	0	0	0.003604
	0	0	0.004751
	0	0	0.005964
	0	0.000296	0.000936
	0	0.000608	0.001922
	0	0.000917	0.002900
	0	0.001238	0.003916
	0	0.001555	0.004918
	0	0.000460	0.000808
	0	0.000924	0.001625
	0	0.001395	0.002452
	0	0.001859	0.003268
	0	0.002334	0.004103
	0	0.000743	0.000527
	0	0.001490	0.001057
	0	0.002220	0.001575
	0	0.002982	0.002115
	0	0.003721	0.002640
	0	0.000942	0.000261
	0	0.001941	0.000537
	0	0.002900	0.000802
	0	0.003832	0.001060
	0	0.004807	0.001329

	0	0.001248	0
	0	0.002465	0
	0	0.003723	0
	0	0.004978	0
	0	0.006244	0
	0	0.000180	0.001131
	0	0.000352	0.002214
	0	0.000527	0.003310
	0	0.000704	0.004427
	0	0.000889	0.005590
	0	0.000355	0.000903
	0	0.000713	0.001817
	0	0.001041	0.002652
	0	0.001405	0.003579
	0	0.001763	0.004490
	0	0.000684	0.000616
	0	0.001371	0.001235
	0	0.002046	0.001842
	0	0.002734	0.002461
	0	0.003426	0.003084
	0	0.000893	0.000465
	0	0.001759	0.000916
	0	0.002650	0.001381
	0	0.003553	0.001852
	0	0.004457	0.002323
	0	0.001100	0.000126
	0	0.002206	0.000252
	0	0.003314	0.000379
	0	0.004422	0.000506
	0	0.005550	0.000635
**Ternary R/SMA/MeCN**	0.000136	0	0.001150
	0.000276	0	0.002330
	0.000416	0	0.003507
	0.000554	0	0.004668
	0.000242	0	0.001049
	0.000490	0	0.002124
	0.000737	0	0.003193
	0.000982	0	0.004254
	0.000354	0	0.000844
	0.000716	0	0.001710
	0.001080	0	0.002579
	0.001440	0	0.003436
	0.000522	0	0.000742

	0.001054	0	0.001499
	0.001587	0	0.002256
	0.002113	0	0.003005
	0.000655	0	0.000649
	0.001316	0	0.001303
	0.001960	0	0.001942
	0.002645	0	0.002620
	0.000663	0	0.000429
	0.001335	0	0.000864
	0.002004	0	0.001296
	0.002676	0	0.001731
	0.003344	0	0.002163
	0.000755	0	0.000319
	0.001523	0	0.000643
	0.002285	0	0.000965
	0.003055	0	0.001290
	0.003813	0	0.001610
	0.000831	0	0.000200
	0.001673	0	0.000403
	0.002518	0	0.000607
	0.003356	0	0.000808
	0.004191	0	0.001010
	0.000942	0	0.000115
	0.001884	0	0.000229
	0.002822	0	0.000344
	0.003764	0	0.000458
	0.004703	0	0.000573
**Quaternary**	0.000492	0.000492	0.000495
	0.000978	0.000978	0.000984
	0.001467	0.001467	0.001476
	0.001950	0.001950	0.001962
	0.000330	0.000330	0.000944
	0.000656	0.000656	0.001874
	0.000981	0.000981	0.002803
	0.001309	0.001309	0.003739
	0.000149	0.000149	0.001218
	0.000297	0.000297	0.002422
	0.000448	0.000448	0.003646
	0.000597	0.000597	0.004859
	0.000678	0.000678	0.000296
	0.001340	0.001340	0.000585
	0.002019	0.002019	0.000881
	0.002682	0.002682	0.001171

	0.000322	0.000970	0.000327
	0.000646	0.001945	0.000656
	0.000985	0.002965	0.001000
	0.001290	0.003884	0.001310
	0.000128	0.000971	0.000154
	0.000257	0.001948	0.000308
	0.000384	0.002911	0.000460
	0.000515	0.003908	0.000618
	0.000642	0.004874	0.000771
	0.000165	0.000830	0.000321
	0.000329	0.001649	0.000639
	0.000491	0.002464	0.000954
	0.000656	0.003292	0.001275
	0.000820	0.004117	0.001595
	0.000123	0.000577	0.000521
	0.000244	0.001148	0.001037
	0.000369	0.001737	0.001569
	0.000495	0.002328	0.002102
	0.000621	0.002922	0.002638
	0.000116	0.000330	0.000794
	0.000234	0.000664	0.001599
	0.000352	0.001001	0.002411
	0.000474	0.001347	0.003242
	0.000595	0.001691	0.004072
	0.000258	0.000522	0.000519
	0.000506	0.001024	0.001018
	0.000760	0.001539	0.001530
	0.001020	0.002064	0.002052
	0.001284	0.002599	0.002584
	0.000442	0.000713	0.000195
	0.000868	0.001400	0.000383
	0.001312	0.002115	0.000579
	0.001746	0.002815	0.000771
	0.002168	0.003495	0.000957
	0.000657	0.000235	0.000218
	0.001320	0.000472	0.000439
	0.001989	0.000711	0.000662
	0.002651	0.000947	0.000882
	0.003310	0.001183	0.001101
	0.000860	0.000113	0.000103
	0.001723	0.000227	0.000206
	0.002594	0.000342	0.000310
	0.003467	0.000457	0.000414

	0.004321	0.000570	0.000516
	0.000680	0.000105	0.000298
	0.001373	0.000213	0.000601
	0.002060	0.000319	0.000902
	0.002750	0.000426	0.001205
	0.003433	0.000532	0.001504
	0.000490	0.000144	0.000475
	0.000989	0.000289	0.000959
	0.001488	0.000436	0.001443
	0.001981	0.000580	0.001921
	0.002474	0.000724	0.002399
	0.000362	0.000098	0.000672
	0.000725	0.000197	0.001347
	0.001092	0.000297	0.002029
	0.001455	0.000396	0.002703
	0.001817	0.000494	0.003375
	0.000448	0.000216	0.000424
	0.000901	0.000435	0.000852
	0.001350	0.000652	0.001276
	0.001803	0.000871	0.001705
	0.002250	0.001087	0.002128
	0.000663	0.000360	0.000110
	0.001338	0.000726	0.000223
	0.002007	0.001090	0.000334
	0.002677	0.001453	0.000445
	0.003343	0.001814	0.000556
	0.000621	0.000207	0.000269
	0.001260	0.000420	0.000546
	0.001891	0.000630	0.000820
	0.002525	0.000842	0.001095
	0.003151	0.001050	0.001366
	0.000825	0.000100	0.000100
	0.001657	0.000201	0.000201
	0.002487	0.000301	0.000301
	0.003320	0.000402	0.000402
	0.004137	0.000501	0.000501
	0.000638	0.000125	0.000314
	0.001279	0.000251	0.000630
	0.001922	0.000377	0.000946
	0.002569	0.000505	0.001264
	0.003207	0.000630	0.001578
	0.000484	0.000106	0.000457
	0.000975	0.000212	0.000920

	0.001463	0.000319	0.001380
	0.001955	0.000426	0.001845
	0.002437	0.000531	0.002298
	0.000308	0.000093	0.000611
	0.000618	0.000187	0.001224
	0.000930	0.000281	0.001842
	0.001240	0.000374	0.002456
	0.001547	0.000467	0.003065
	0.000389	0.000194	0.000405
	0.000787	0.000392	0.000818
	0.001189	0.000592	0.001236
	0.001586	0.000790	0.001650
	0.001980	0.000987	0.002059
	0.000640	0.000349	0.000094
	0.001269	0.000693	0.000187
	0.001922	0.001049	0.000282
	0.002560	0.001397	0.000376
	0.003197	0.001744	0.000470

S2.2 – Models validation by comparing results with gravimetric method

Table S2: Comparison of results obtained from UV-CD calibration method and gravimetry on the same 28 solutions of varying compositions in S and S-MA. The percentage error $\delta\ (\%) = \frac{|X-Y|}{Y}$ is used, with X being the total solubility obtained with UV-CD models result, and Y the total solubility from gravimetric method, on the same saturated solution.

**Mass fraction total solid *X* (UV-CD)**	**Mass fraction total solid *Y* (Gravimetry)**	**Percentage Error *δ* (%)**
0.037	0.037	0.294
0.038	0.038	0.198
0.040	0.039	0.361
0.138	0.133	3.528
0.139	0.137	1.912
0.135	0.132	2.168
0.213	0.214	0.459
0.224	0.227	1.261
0.216	0.214	0.567
0.236	0.234	0.725
0.245	0.241	1.583
0.240	0.236	1.982
0.258	0.254	1.444
0.270	0.288	6.368
0.099	0.095	4.283
0.105	0.101	3.634
0.117	0.114	2.463
0.110	0.107	2.341
0.125	0.123	2.206
0.118	0.116	1.599
0.114	0.113	0.861
0.114	0.111	2.636
0.114	0.112	2.136
0.128	0.122	4.468
0.118	0.121	2.507
0.135	0.13	3.881
0.140	0.136	2.507
0.152	0.152	0.192
**Average percentage error *δ***		**2.091**
**Standard deviation**		**1.472**

S3 – Phase diagrams data

Table S2: Solubilities of pure solid phases, eutectic points in ternary sections and quaternary points from the quaternary system R/S/S-MA/MeCN results at 9°C. Pure R, S and S-MA solubilities are the average of four compositions points. Pure RS and pure S:S-MA solubilities are computed from extrapolation of solubility curves. Points a, b and c were found experimentally. Points d, e and f were estimated from intersection of neighbouring solubility curves or surfaces. Points g and h could not be determined because of very high viscosity of saturated solutions in the region they belong, therefore only a roughly estimated compositional region is proposed.

Solubility at 9°C	Molar fraction X in dissolved components (%mol)	Mass concentration (mg/mL MeCN)
Pure R and S	**R/S:** 0.933	**R/S:** 30.7
Pure RS	**R:** 0.302 **S:** 0.302<br>**Total:** 0.604	**R:** 9.9 **S:** 9.9<br>**Total:** 19.8
Pure S-MA (average)	**S-MA:** 4.054	**S-MA:** 123.2
Pure S:S-MA	**S:** 1.591 **S-MA:** 1.591<br>**Total:** 3.182	**S:** 53.6 **S-MA:** 47.9<br>**Total:** 101.5
Eutectic point ***a***<br>(equilibrium with R and RS)	**R:** 0.954 **S:** 0.134<br>**Total:** 1.088	**R:** 31.4 **S:** 4.4<br>**Total:** 35.8
Eutectic point ***b***<br>(equilibrium with RS and S)	**R:** 0.134 **S:** 0.954<br>**Total:** 1.088	**R:** 4.4 **S:** 31.4<br>**Total:** 35.8
Eutectic point ***c***<br>(equilibrium with S and S:S-MA)	**S:** 1.974 **S-MA:** 1.253<br>**Total:** 3.227	**S:** 66.5 **S-MA:** 37.7<br>**Total:** 102.2
Eutectic point ***d***<br>(equilibrium with S:S-MA and S-MA)	**S:** 1.799 **S-MA:** 7.132<br>**Total:** 8.931	**S:** 64.4 **S-MA:** 228.2<br>**Total:** 292.6
Eutectic point ***e***<br>(equilibrium with R and S-MA)	**R:** 29.5 **S-MA:** 36.5<br>**Total:** 66.0	**R:** 2827.7 **S-MA:** 3127.4<br>**Total:** 5955.1
Quaternary point ***f***<br>(equilibrium with S, RS and S:S-MA)	**R:** 0.356 **S:** 2.176 **S-MA:** 1.513<br>**Total:** 4.045	**R:** 12.1 **S:** 73.9 **S-MA:** 45.9<br>**Total:** 131.9

Quaternary point ***g*** (equilibrium with R, RS and S-MA)	**Solvent-free ratio**<br>**R:** 38.1% **S:** 8.1% **S-MA:** 53.8%	Total solubility > 6000 mg/mL MeCN
Quaternary point ***h*** (equilibrium with RS, S-MA and S:S-MA)	**Solvent-free ratio**<br>**R:** 43.0% **S:** 6.0% **S-MA:** 51.0%	Total solubility > 6000 mg/mL MeCN

S3.1 – Isothermal ternary system between enantiomers R and S

Table S4: Data used for the ternary phase diagram R/S/MeCN at 9°C.

**Model prediction from UV-CD**			**Dilution**	**Final liquid composition (molar fraction)**			**Solid**
x_{S+R} of diluted solution	x_{S-R} of diluted solution	Enantiomeric excess *E* of diluted solution (%)	Dilution ratio	X_R (%)	X_S (%)	X_{MeCN} (%)	Solid phase(s) in suspension (XRPD)
0.00336	0.00336	100.00	10.667	0.000	0.889	99.111	S
0.00333	0.00310	93.07	12.045	0.035	0.964	99.002	S
0.00369	0.00328	88.78	10.573	0.054	0.916	99.029	S
0.00375	0.00320	85.41	10.643	0.072	0.920	99.008	S
0.00380	0.00300	78.94	11.104	0.111	0.942	98.947	S
0.00396	0.00302	76.16	10.915	0.129	0.950	98.922	S
0.00385	0.00290	75.33	11.335	0.134	0.954	98.911	S + RS
0.00396	0.00289	73.08	10.913	0.145	0.933	98.922	RS
0.00372	0.00265	71.14	10.813	0.144	0.857	98.999	RS
0.00351	0.00240	68.49	11.213	0.154	0.825	99.021	RS
0.00360	0.00248	68.98	11.040	0.153	0.835	99.012	RS
0.00287	0.00187	65.08	12.698	0.158	0.745	99.097	RS
0.00261	0.00170	65.09	12.930	0.146	0.689	99.165	RS
0.00331	0.00209	63.05	10.922	0.166	0.731	99.103	RS
0.00281	0.00163	57.92	12.332	0.180	0.677	99.143	RS
0.00287	0.00155	54.11	11.431	0.186	0.625	99.189	RS
0.00234	0.00129	55.02	11.603	0.150	0.518	99.332	RS
0.00255	0.00123	48.17	12.401	0.202	0.578	99.220	RS
0.00257	0.00109	42.52	11.329	0.207	0.512	99.281	RS
0.00240	0.00096	40.10	11.116	0.197	0.460	99.343	RS
0.00230	0.00075	32.68	11.276	0.214	0.422	99.363	RS
0.00232	0.00063	27.11	10.926	0.227	0.395	99.378	RS
0.00218	0.00040	18.42	11.430	0.250	0.363	99.387	RS
0.00229	0.00038	16.50	10.825	0.255	0.355	99.390	RS
0.00191	0.00018	9.50	12.109	0.257	0.311	99.432	RS
0.00212	0	0	11.614	0.302	0.302	99.396	RS

The 26 other points corresponding to excess of R were deducted from mirror projection due to symmetry along racemic compositions in enantiomeric systems

Table S5: Comparison of four solubility values variation for pure R and S solubilities and S-MA with UV-CD spectroscopy and gravimetry methods.

	UV-CD spectroscopy		Gravimetry
	***X* (% mol)**	**Concentration (mg/mL)**	
**Pure R and S**	0.889	29.218	-
	0.924	30.383	30.438
	0.935	30.756	30.981
	0.986	32.457	32.207
**Average**	**0.933**	**30.703**	**31.209**
**Standard deviation**	0.035	1.161	0.734
**Pure S-MA**	4.103	124.699	-
	4.076	123.861	121.052
	4.054	123.251	124.604
	3.985	120.961	119.771
**Average**	**4.054**	**123.193**	**121.809**
**Standard deviation**	0.044	1.387	2.044

S3.2 – Isothermal ternary system between enantiomer S and S-SMA

Table S6: Data used for the ternary phase diagram S/S-MA/MeCN at 9°C.

**Model prediction from UV-CD**		**Dilution**	**Final liquid composition (molar fraction)**			**Solid**
x_S in diluted solution	$x_{S\text{-}MA}$ in diluted solution	Dilution ratio	X_S (%)	$X_{S\text{-}MA}$ (%)	X_{MeCN} (%)	Solid phase(s) in suspension (XRPD)
0.00403	0.00000	9.237	0.924	0.000	99.083	S
0.00405	0.00122	11.974	1.229	0.414	98.357	S
0.00462	0.00185	11.946	1.412	0.633	97.954	S
0.00295	0.00146	20.953	1.603	0.887	97.509	S
0.00307	0.00158	20.601	1.641	0.944	97.415	S
0.00172	0.00000	21.678	0.924	0.000	99.071	S
0.00173	0.00000	21.764	0.935	0.000	99.060	S
0.00186	0.00000	21.381	0.986	0.000	99.018	S
0.00096	0.00050	67.722	1.701	0.983	97.316	S
0.00105	0.00052	66.641	1.833	1.016	97.152	S
0.00043	0.00378	42.511	0.502	4.984	94.514	S-MA
0.00028	0.00331	45.989	0.351	4.667	94.982	S-MA
0.00019	0.00256	58.420	0.307	4.575	95.118	S-MA

0.00003	0.00216	63.399	0.045	4.103	95.853	S-MA
0.00004	0.00212	63.946	0.063	4.076	95.861	S-MA
0.00006	0.00173	78.056	0.121	4.054	95.825	S-MA
0.00003	0.00204	65.176	0.053	3.984	95.962	S-MA
0.00054	0.00307	59.035	0.903	5.786	93.311	S-MA
0.00064	0.00316	58.974	1.082	6.026	92.892	S-MA
0.00059	0.00299	60.309	1.016	5.778	93.206	S-MA
0.00097	0.00420	45.585	1.293	6.257	92.449	S-MA
0.00074	0.00303	64.972	1.405	6.486	92.109	S-MA
0.00074	0.00315	61.701	1.338	6.379	92.283	S-MA
0.00105	0.00396	51.485	1.603	6.786	91.611	S-MA
0.00098	0.00359	59.026	1.739	7.132	91.130	S-MA
0.00101	0.00104	49.702	1.317	1.512	97.171	S:S-MA
0.00101	0.00244	52.329	1.477	3.981	94.542	S:S-MA
0.00078	0.00215	62.629	1.369	4.207	94.423	S:S-MA
0.00088	0.00253	56.878	1.414	4.530	94.055	S:S-MA
0.00106	0.00327	47.867	1.446	4.990	93.564	S:S-MA
0.00120	0.00371	42.160	1.444	4.980	93.576	S:S-MA
0.00110	0.00345	46.829	1.476	5.164	93.360	S:S-MA
0.00090	0.00287	57.190	1.479	5.271	93.250	S:S-MA
0.00114	0.00372	46.580	1.534	5.617	92.849	S:S-MA
0.00119	0.00423	44.496	1.554	6.169	92.278	S:S-MA
0.00119	0.00422	47.378	1.678	6.646	91.676	S:S-MA
0.00102	0.00360	56.960	1.733	6.864	91.403	S:S-MA
0.00119	0.00421	48.618	1.726	6.842	91.432	S:S-MA
0.00100	0.00352	58.655	1.766	6.930	91.304	S:S-MA
0.00129	0.00454	45.573	1.764	6.947	91.289	S:S-MA
0.00109	0.00358	49.021	1.545	5.694	92.761	S:S-MA
0.00112	0.00367	46.113	1.491	5.456	93.053	S:S-MA
0.00116	0.00401	48.195	1.650	6.380	91.970	S:S-MA
0.00130	0.00089	49.903	1.710	1.309	96.981	S:S-MA
0.00125	0.00075	62.757	2.086	1.397	96.516	S:S-MA
0.00104	0.00069	67.882	1.872	1.386	96.741	S:S-MA
0.00102	0.00074	64.592	1.735	1.409	96.855	S:S-MA
0.00087	0.00079	68.930	1.575	1.608	96.817	S:S-MA
0.00085	0.00094	63.791	1.425	1.768	96.807	S:S-MA
0.00081	0.00107	68.101	1.464	2.174	96.361	S:S-MA
0.00073	0.00117	61.931	1.191	2.142	96.666	S:S-MA
0.00092	0.00165	52.348	1.297	2.596	96.107	S:S-MA
0.00077	0.00156	60.033	1.239	2.816	95.946	S:S-MA
0.00080	0.00178	58.983	1.279	3.190	95.532	S:S-MA
0.00117	0.00066	63.977	1.974	1.253	96.773	S + S:S-MA

S3.3 – Isothermal ternary system between enantiomer R and S-MA

Table S7: Data used for the ternary phase diagram R/S-MA/MeCN at 9°C.

**Model prediction from UV-CD**		**Dilution**	**Final liquid composition (molar fraction)**			**Solid**
x_R in diluted solution	$x_{S\text{-}MA}$ in diluted solution	Dilution ratio	X_R (%)	$X_{S\text{-}MA}$ (%)	X_{MeCN} (%)	Solid phase(s) in suspension (XRPD)
0.00094	0.00011	53.036	1.262	0.168	98.571	R
0.00109	0.00026	51.026	1.411	0.379	98.211	R
0.00091	0.00036	89.046	2.133	0.935	96.932	R
0.00103	0.00047	87.759	2.424	1.240	96.336	R
0.00124	0.00072	83.848	2.860	1.865	95.274	R
0.00079	0.00048	163.125	3.695	2.477	93.827	R
0.00130	0.00088	110.166	4.209	3.199	92.592	R
0.00122	0.00086	133.803	4.968	3.940	91.092	R
0.00101	0.00079	178.077	5.719	4.967	89.313	R
0.00133	0.00106	153.884	6.825	6.054	87.121	R
0.00150	0.00125	143.384	7.332	6.861	85.808	R
0.00117	0.00103	190.982	7.878	7.737	84.385	R
0.00125	0.00116	214.444	10.525	10.937	78.537	R
0.00128	0.00109	231.981	12.150	11.580	76.270	R
0.00126	0.00106	246.714	13.073	12.280	74.647	R
0.00151	0.00142	196.486	12.486	13.146	74.368	R
0.00155	0.00139	210.004	14.561	14.624	70.815	R
0.00150	0.00138	225.100	15.800	16.276	67.925	R
0.00142	0.00129	229.829	14.755	14.985	70.260	R
0.00078	0.00147	218.602	6.469	13.668	79.862	S-MA
0.00068	0.00135	234.158	5.934	13.177	80.889	S-MA
0.00071	0.00161	180.174	4.469	11.307	84.224	S-MA
0.00083	0.00214	124.180	3.413	9.862	86.726	S-MA
0.00053	0.00149	163.260	2.760	8.686	88.554	S-MA
0.00062	0.00222	95.137	1.777	7.096	91.127	S-MA
0.00050	0.00207	93.682	1.363	6.344	92.294	S-MA
0.00029	0.00275	59.964	0.491	5.127	94.382	S-MA
0.00017	0.00242	65.795	0.308	4.911	94.781	S-MA
			**Suspension composition**			
			19.914	26.145	53.942	No solid
			22.539	29.862	47.599	No solid
			24.740	32.748	42.511	S-MA
			26.783	35.136	38.080	S-MA
			29.847	39.608	30.545	S-MA

S3.4 – Quaternary phase diagram R/S/S-MA/MeCN

Table S8: Data used for the quaternary phase diagram R/S/S-MA/MeCN at 9°C (inside of the tetrahedron).

Model prediction from UV-CD			Dilution	Final liquid composition (molar fraction)				Solubility surface projection (molar fraction)			Solid phase(s) in suspension (XRPD)
x_R in diluted solution	x_S in diluted solution	$x_{S\text{-}MA}$ in diluted solution	Dilution ratio	X_R (%)	X_S (%)	$X_{S\text{-}MA}$ (%)	X_{MeCN} (%)	X_S (%)	$X_{S\text{-}MA}$ (%)	X_R (%)	XRPD
0.00087	0.00087	0.00177	48.943	1.171	1.174	2.675	94.980	23.386	53.281	23.334	RS
0.00055	0.00052	0.00102	70.578	1.045	1.002	2.179	95.774	23.706	51.572	24.723	RS
0.00041	0.00038	0.00066	76.957	0.828	0.773	1.493	96.906	24.983	48.256	26.761	RS
0.00062	0.00057	0.00094	42.213	0.679	0.625	1.152	97.544	25.458	46.895	27.647	RS
0.00046	0.00050	0.00050	42.664	0.498	0.538	0.599	98.365	32.895	36.643	30.462	RS
0.00020	0.00116	0.00050	46.746	0.237	1.402	0.676	97.685	60.564	29.215	10.221	S+RS
0.00020	0.00120	0.00042	40.499	0.212	1.241	0.483	98.065	64.121	24.932	10.948	S+RS
0.00020	0.00125	0.00044	38.312	0.194	1.224	0.485	98.098	64.332	25.488	10.181	S+RS
0.00018	0.00131	0.00045	37.799	0.177	1.267	0.491	98.064	65.477	25.381	9.141	S+RS
0.00026	0.00154	0.00080	39.679	0.274	1.602	0.927	97.198	57.169	33.065	9.766	S+RS
0.00027	0.00169	0.00095	38.027	0.268	1.692	1.068	96.972	55.871	35.275	8.854	S+RS
0.00021	0.00144	0.00074	43.277	0.234	1.632	0.932	97.201	58.321	33.307	8.372	S+RS
0.00020	0.00122	0.00077	62.745	0.336	2.050	1.455	96.159	53.376	37.875	8.748	S+RS
0.00021	0.00131	0.00081	55.686	0.310	1.947	1.347	96.397	54.032	37.377	8.591	S+RS
0.00022	0.00135	0.00088	53.674	0.317	1.942	1.408	96.333	52.964	38.385	8.651	S:S-MA+S
0.00018	0.00093	0.00069	74.849	0.364	1.860	1.539	96.237	49.426	40.895	9.678	S:S-MA+RS
0.00020	0.00110	0.00068	68.658	0.377	2.023	1.411	96.190	53.092	37.023	9.885	S:S-MA
0.00016	0.00119	0.00073	63.320	0.264	2.019	1.379	96.338	55.140	37.658	7.201	S:S-MA
0.00029	0.00089	0.00090	65.956	0.515	1.575	1.785	96.125	40.648	46.059	13.293	S:S-MA
0.00021	0.00099	0.00082	64.593	0.355	1.704	1.587	96.355	46.744	43.521	9.735	S:S-MA
0.00071	0.00090	0.00259	59.571	1.259	1.589	5.118	92.034	19.944	64.247	15.809	S:S-MA
0.00041	0.00093	0.00210	59.825	0.700	1.589	4.000	93.711	25.275	63.601	11.123	S:S-MA
0.00035	0.00081	0.00289	65.234	0.691	1.590	6.315	91.404	18.502	73.458	8.040	S:S-MA

0.00040	0.00106	0.00398	54.905	0.688	1.799	7.561	89.952	17.909	75.247	6.844	S:S-MA
0.00038	0.00041	0.00330	57.816	0.649	0.691	6.227	92.434	9.131	82.297	8.572	S-MA
0.00064	0.00068	0.00377	60.716	1.211	1.295	7.999	89.494	12.328	76.140	11.531	S-MA
0.00090	0.00096	0.00333	65.173	1.882	2.005	7.809	88.305	17.143	66.769	16.088	S:S-MA
0.00150	0.00122	0.00369	48.852	2.303	1.877	6.336	89.484	17.847	60.253	21.900	S:S-MA
0.00024	0.00094	0.00033	39.949	0.238	0.951	0.369	98.443	61.046	23.689	15.265	RS
0.00022	0.00140	0.00039	38.838	0.221	1.395	0.437	97.947	67.927	21.304	10.768	S+RS
0.00024	0.00132	0.00035	38.104	0.231	1.286	0.383	98.100	67.666	20.158	12.177	S+RS
0.00020	0.00129	0.00038	40.246	0.209	1.322	0.440	98.028	67.071	22.328	10.601	S+RS
0.00020	0.00140	0.00047	36.829	0.188	1.321	0.495	97.995	65.905	24.699	9.396	S
0.00032	0.00132	0.00055	43.854	0.370	1.510	0.705	97.415	58.421	27.269	14.310	RS
0.00034	0.00110	0.00079	39.231	0.349	1.110	0.890	97.651	47.265	37.885	14.850	RS
0.00028	0.00160	0.00068	38.293	0.278	1.595	0.759	97.368	60.604	28.837	10.559	RS
0.00024	0.00163	0.00080	40.602	0.256	1.735	0.953	97.057	58.935	32.374	8.691	S
0.00042	0.00160	0.00106	35.765	0.394	1.506	1.118	96.982	49.913	37.030	13.057	RS
0.00029	0.00180	0.00112	44.681	0.356	2.176	1.513	95.955	53.799	37.400	8.802	S+RS
0.00062	0.00131	0.00139	40.116	0.667	1.412	1.672	96.249	37.645	44.577	17.778	RS
0.00041	0.00217	0.00137	36.006	0.394	2.110	1.487	96.009	52.871	37.252	9.876	RS
0.00026	0.00200	0.00119	39.848	0.274	2.147	1.426	96.153	55.807	37.064	7.129	S:S-MA+S
0.00034	0.00095	0.00091	63.642	0.581	1.626	1.753	96.040	41.070	44.258	14.671	RS
0.00034	0.00188	0.00116	41.019	0.371	2.075	1.427	96.127	53.574	36.851	9.575	S:S-MA+RS
0.00065	0.00094	0.00141	52.943	0.937	1.364	2.284	95.415	29.749	49.808	20.444	RS
0.00034	0.00057	0.00101	65.902	0.594	0.999	1.988	96.419	27.906	55.507	16.587	RS
0.00078	0.00106	0.00184	52.099	1.146	1.560	3.012	94.282	27.285	52.674	20.041	RS
0.00068	0.00108	0.00171	55.322	1.061	1.689	2.983	94.267	29.461	52.038	18.502	S:S-MA
0.00024	0.00102	0.00136	53.308	0.348	1.457	2.181	96.015	36.563	54.714	8.723	S:S-MA
0.00094	0.00114	0.00227	49.959	1.344	1.631	3.647	93.379	24.628	55.080	20.292	S:S-MA
0.00092	0.00106	0.00334	51.472	1.432	1.654	5.816	91.098	18.582	65.330	16.088	S:S-MA
0.00057	0.00108	0.00389	43.588	0.725	1.377	5.562	92.337	17.968	72.575	9.457	S:S-MA
0.00112	0.00122	0.00398	49.605	1.742	1.896	6.928	89.434	17.948	65.565	16.487	S:S-MA
0.00077	0.00098	0.00408	53.499	1.285	1.644	7.643	89.428	15.546	72.297	12.157	S:S-MA

0.00057	0.00107	0.00468	51.981	0.937	1.777	8.657	88.628	15.630	76.126	8.244	S-MA
0.00054	0.00102	0.00469	47.279	0.780	1.491	7.641	90.087	15.043	77.085	7.872	S-MA
0.00028	0.00012	0.00117	139.523	1.109	0.472	5.273	93.145	6.892	76.927	16.181	S-MA
0.00045	0.00013	0.00158	141.129	1.971	0.575	7.755	89.699	5.581	75.285	19.133	S-MA
0.00037	0.00018	0.00152	146.910	1.673	0.837	7.773	89.717	8.141	75.587	16.272	S-MA
0.00072	0.00027	0.00237	112.468	2.714	1.015	9.967	86.304	7.412	72.772	19.817	S-MA
0.00039	0.00024	0.00142	181.283	2.359	1.430	9.535	86.675	10.734	71.560	17.706	S-MA
0.00058	0.00018	0.00153	189.884	3.945	1.205	11.554	83.296	7.214	69.170	23.616	S-MA
0.00050	0.00024	0.00145	197.506	3.517	1.672	11.368	83.444	10.096	68.663	21.241	S-MA
0.00047	0.00033	0.00169	170.410	2.784	1.999	11.301	83.915	12.431	70.260	17.309	S-MA
0.00079	0.00032	0.00193	184.459	6.030	2.456	16.468	75.046	9.843	65.994	24.163	S-MA
0.00060	0.00036	0.00162	223.500	5.683	3.373	16.999	73.945	12.945	65.242	21.813	S-MA
0.00046	0.00031	0.00115	257.884	4.582	3.097	12.746	79.576	15.162	62.404	22.434	S:S-MA
0.00084	0.00033	0.00142	252.583	9.991	3.924	18.901	67.184	11.958	57.596	30.447	S:S-MA
0.00097	0.00042	0.00186	188.424	8.122	3.544	17.382	70.952	12.199	59.840	27.962	S:S-MA
0.00097	0.00050	0.00202	157.046	6.177	3.195	14.408	76.220	13.436	60.589	25.975	S:S-MA
0.00073	0.00045	0.00141	169.992	4.461	2.736	9.650	83.153	16.242	57.281	26.478	S:S-MA
0.00110	0.00044	0.00172	190.280	9.336	3.735	16.402	70.527	12.673	55.650	31.677	S:S-MA
0.00098	0.00039	0.00168	195.249	8.317	3.272	15.876	72.534	11.915	57.804	30.281	S:S-MA
0.00079	0.00037	0.00151	193.166	5.986	2.813	12.718	78.483	13.074	59.109	27.818	S:S-MA
0.00056	0.00036	0.00121	211.423	4.250	2.744	10.307	82.698	15.862	59.575	24.563	S:S-MA
0.00123	0.00039	0.00162	189.678	10.394	3.252	15.297	71.056	11.237	52.852	35.911	RS
0.00098	0.00036	0.00144	214.768	9.137	3.376	14.960	72.527	12.287	54.455	33.258	S:S-MA
0.00105	0.00042	0.00158	190.103	8.416	3.356	14.221	74.007	12.913	54.709	32.379	S:S-MA
0.00086	0.00044	0.00140	163.365	5.079	2.591	9.221	83.109	15.338	54.595	30.067	S:S-MA
0.00069	0.00038	0.00111	162.698	3.695	2.035	6.598	87.671	16.506	53.521	29.973	S:S-MA
0.00150	0.00032	0.00169	145.224	8.476	1.818	10.671	79.036	8.672	50.899	40.429	No solid
0.00145	0.00041	0.00187	121.134	6.392	1.797	9.197	82.615	10.334	52.901	36.765	RS
0.00090	0.00040	0.00137	155.469	4.861	2.185	8.282	84.672	14.253	54.033	31.714	RS
0.00062	0.00034	0.00099	165.274	3.276	1.778	5.808	89.138	16.371	53.471	30.159	S:S-MA+RS
0.00045	0.00045	0.00014	39.876	0.448	0.442	0.160	98.950	42.131	15.194	42.674	RS

0.00038	0.00038	0.00024	53.510	0.509	0.508	0.361	98.622	36.854	26.195	36.951	RS
0.00051	0.00047	0.00032	45.035	0.574	0.536	0.407	98.483	35.312	26.854	37.834	RS
0.00063	0.00059	0.00077	45.195	0.735	0.684	1.010	97.570	28.166	41.575	30.259	RS
0.00057	0.00054	0.00077	48.477	0.714	0.679	1.085	97.521	27.403	43.779	28.818	RS
0.00068	0.00066	0.00107	46.267	0.827	0.808	1.460	96.905	26.099	47.170	26.732	RS
0.00027	0.00089	0.00019	44.731	0.302	1.000	0.237	98.461	64.989	15.377	19.634	RS
0.00020	0.00094	0.00010	47.574	0.242	1.132	0.140	98.485	74.722	9.273	16.005	RS
0.00017	0.00108	0.00017	44.796	0.192	1.224	0.221	98.363	74.761	13.504	11.734	S+RS
0.00017	0.00108	0.00018	45.541	0.192	1.243	0.229	98.336	74.708	13.783	11.509	S+RS
0.00015	0.00107	0.00008	48.212	0.189	1.305	0.103	98.404	81.751	6.431	11.818	S+RS
0.00017	0.00116	0.00017	47.046	0.203	1.390	0.230	98.176	76.237	12.624	11.139	S+RS
0.00015	0.00104	0.00010	45.235	0.170	1.190	0.125	98.515	80.129	8.406	11.465	S+RS
0.00017	0.00110	0.00014	44.706	0.188	1.245	0.178	98.389	77.260	11.076	11.664	S+RS
0.00012	0.00098	0.00011	49.294	0.150	1.218	0.159	98.473	79.770	10.398	9.832	S
0.00015	0.00125	0.00042	45.802	0.173	1.469	0.559	97.800	66.759	25.394	7.847	S
0.00010	0.00149	0.00065	47.349	0.118	1.846	0.903	97.133	64.380	31.500	4.121	S
0.00011	0.00171	0.00083	45.177	0.126	2.043	1.106	96.725	62.379	33.780	3.841	S
0.00012	0.00171	0.00103	47.552	0.153	2.188	1.475	96.183	57.339	38.652	4.009	S
0.00018	0.00132	0.00032	44.499	0.202	1.512	0.415	97.871	71.023	19.491	9.486	S+RS
0.00036	0.00077	0.00046	45.547	0.414	0.899	0.599	98.088	47.026	31.342	21.632	RS
0.00026	0.00138	0.00073	42.597	0.285	1.536	0.906	97.273	56.316	33.215	10.469	RS
0.00046	0.00088	0.00090	45.994	0.555	1.054	1.212	97.179	37.365	42.972	19.663	RS
0.00016	0.00169	0.00112	51.500	0.219	2.363	1.757	95.661	54.454	40.500	5.046	S:S-MA+S
0.00012	0.00160	0.00106	52.515	0.175	2.274	1.687	95.865	54.980	40.794	4.226	S:S-MA+S
0.00015	0.00179	0.00129	49.160	0.200	2.411	1.937	95.452	53.018	42.590	4.392	S:S-MA+S
0.00015	0.00187	0.00137	49.325	0.211	2.537	2.082	95.171	52.527	43.110	4.363	S:S-MA+S
0.00011	0.00185	0.00134	51.247	0.158	2.623	2.121	95.098	53.514	43.273	3.214	S:S-MA+S
0.00104	0.00014	0.00034	54.444	1.458	0.202	0.528	97.812	9.211	24.135	66.654	R+RS
0.00110	0.00018	0.00026	48.083	1.355	0.216	0.355	98.074	11.212	18.452	70.336	R+RS
0.00098	0.00016	0.00028	52.079	1.310	0.216	0.423	98.051	11.108	21.680	67.212	R+RS
0.00090	0.00021	0.00021	48.209	1.095	0.256	0.288	98.360	15.636	17.574	66.790	RS

0.00090	0.00013	0.00051	89.075	2.150	0.322	1.364	96.163	8.396	35.563	56.040	R+RS
0.00060	0.00012	0.00029	108.553	1.714	0.355	0.927	97.004	11.841	30.936	57.223	R+RS
0.00066	0.00015	0.00029	97.926	1.686	0.384	0.842	97.088	13.202	28.903	57.895	R+RS
0.00056	0.00016	0.00033	93.800	1.359	0.388	0.893	97.360	14.684	33.836	51.480	RS
0.00085	0.00013	0.00046	99.596	2.276	0.361	1.392	95.972	8.959	34.552	56.490	R+RS
0.00084	0.00014	0.00047	99.598	2.256	0.367	1.400	95.977	9.120	34.800	56.080	R+RS
0.00099	0.00016	0.00057	82.983	2.216	0.351	1.432	96.001	8.777	35.802	55.421	R+RS
0.00086	0.00022	0.00068	54.739	1.228	0.311	1.081	97.380	11.875	41.265	46.860	RS
0.00080	0.00013	0.00057	132.948	3.011	0.472	2.389	94.128	8.033	40.689	51.278	R+RS
0.00077	0.00016	0.00058	140.770	3.089	0.633	2.640	93.638	9.950	41.496	48.554	R+RS
0.00073	0.00013	0.00054	140.845	2.898	0.535	2.422	94.144	9.144	41.362	49.494	RS
0.00103	0.00015	0.00085	121.991	3.701	0.526	3.430	92.344	6.867	44.794	48.339	R+RS
0.00090	0.00014	0.00066	151.953	4.063	0.641	3.348	91.948	7.965	41.576	50.459	R+RS
0.00106	0.00020	0.00086	119.881	3.774	0.717	3.453	92.057	9.022	43.469	47.509	RS
0.00102	0.00013	0.00081	167.964	5.469	0.672	4.889	88.970	6.096	44.323	49.581	R+RS
0.00091	0.00014	0.00074	183.369	5.339	0.850	4.863	88.948	7.691	44.003	48.306	RS
0.00068	0.00036	0.00097	142.777	2.993	1.560	4.737	90.710	16.791	50.993	32.217	RS
0.00115	0.00014	0.00099	171.902	6.745	0.793	6.451	86.012	5.668	46.115	48.217	R+RS
0.00069	0.00011	0.00059	287.405	6.847	1.125	6.589	85.440	7.724	45.252	47.024	RS
0.00058	0.00022	0.00074	243.135	4.702	1.807	6.704	86.786	13.677	50.736	35.587	RS
0.00115	0.00015	0.00108	184.329	7.575	1.018	8.017	83.390	6.130	48.268	45.602	RS
0.00085	0.00025	0.00103	202.866	6.135	1.811	8.288	83.766	11.153	51.055	37.792	RS
0.00154	0.00021	0.00161	173.935	11.413	1.575	13.348	73.664	5.982	50.683	43.335	R+RS
0.00103	0.00027	0.00126	189.507	7.360	1.937	10.053	80.650	10.012	51.952	38.036	RS
0.00072	0.00057	0.00105	85.093	1.722	1.361	2.829	94.088	23.018	47.854	29.128	RS
0.00071	0.00057	0.00119	84.666	1.720	1.376	3.220	93.684	21.790	50.982	27.228	S:S-MA+RS
0.00078	0.00061	0.00122	95.143	2.202	1.712	3.843	92.243	22.070	49.543	28.387	RS
0.00102	0.00075	0.00168	75.951	2.316	1.713	4.278	91.694	20.620	51.499	27.881	S:S-MA+RS
0.00078	0.00049	0.00136	142.924	3.740	2.338	7.280	86.642	17.500	54.504	27.996	S:S-MA
0.00079	0.00046	0.00124	168.396	4.641	2.740	8.191	84.428	17.594	52.601	29.804	S:S-MA+RS
0.00076	0.00040	0.00162	159.830	4.356	2.315	10.423	82.906	13.542	60.973	25.485	S:S-MA

0.00080	0.00050	0.00167	159.326	4.713	2.989	11.069	81.229	15.924	58.968	25.109	S:S-MA
0.00088	0.00066	0.00265	109.643	3.527	2.645	11.883	81.944	14.652	65.813	19.536	S:S-MA
0.00093	0.00068	0.00285	103.072	3.513	2.555	12.016	81.916	14.130	66.447	19.423	S:S-MA+S-MA
0.00045	0.00037	0.00224	97.896	1.362	1.136	7.598	89.903	11.254	75.255	13.491	S-MA
0.00038	0.00032	0.00193	106.492	1.231	1.021	6.987	90.761	11.050	75.623	13.327	S-MA
0.00007	0.00011	0.00157	107.460	0.211	0.333	5.280	94.176	5.711	90.657	3.631	S-MA
0.00036	0.00013	0.00176	103.977	1.078	0.394	5.963	92.565	5.295	80.203	14.501	S-MA
0.00071	0.00046	0.00107	113.836	2.413	1.552	4.057	91.977	19.349	50.571	30.080	S:S-MA+RS
0.00087	0.00050	0.00130	109.018	2.926	1.674	4.882	90.518	17.650	51.493	30.857	S:S-MA+RS
0.00098	0.00058	0.00151	100.426	3.086	1.819	5.311	89.785	17.804	51.989	30.207	S:S-MA+RS
0.00113	0.00056	0.00174	118.247	4.626	2.277	7.952	85.145	15.330	53.527	31.142	S:S-MA+RS
0.00113	0.00043	0.00160	141.261	5.760	2.199	9.132	82.909	12.866	53.432	33.702	RS
0.00124	0.00036	0.00167	210.002	12.849	3.724	19.352	64.075	10.365	53.867	35.767	S:S-MA
0.00114	0.00029	0.00153	236.452	13.571	3.445	20.367	62.618	9.215	54.482	36.303	S:S-MA
0.00138	0.00038	0.00174	216.332	16.505	4.545	23.281	55.668	10.252	52.517	37.231	S:S-MA
0.00134	0.00035	0.00183	200.696	13.701	3.540	20.886	61.873	9.285	54.779	35.936	S:S-MA
0.00092	0.00035	0.00123	290.681	13.914	5.246	20.925	59.914	13.088	52.201	34.711	S:S-MA
0.00123	0.00047	0.00176	203.680	12.722	4.840	20.341	62.097	12.769	53.665	33.566	S:S-MA
0.00086	0.00036	0.00124	283.132	12.203	5.098	19.672	63.028	13.789	53.207	33.005	S:S-MA
0.00148	0.00035	0.00186	211.656	17.917	4.191	25.276	52.616	8.844	53.343	37.813	S:S-MA
0.00140	0.00036	0.00191	195.214	14.113	3.629	21.540	60.717	9.238	54.834	35.927	S:S-MA

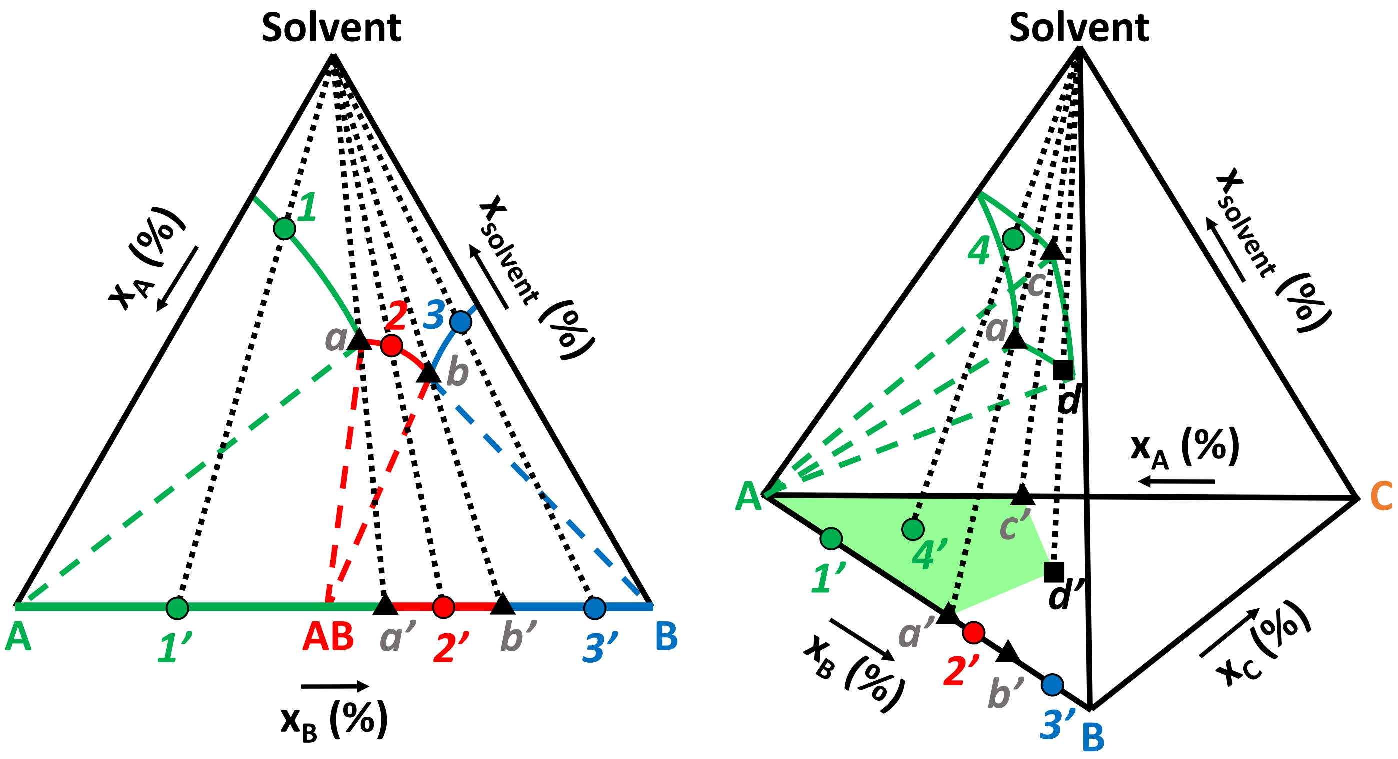

Figure S3: Left: projection of solubility curves (solid lines) of A (green), AB (red) and B (blue) on solvent-free axis AB in the isothermal ternary phase diagram A/B/Solvent. Dotted lines connect solubility compositions (1, 2, 3, a, b) to their solvent-free equivalent (1', 2', 3', a', b'). Dashed lines represent the boundaries of phase stability domains. Eutectic compositions a and b (black triangles) represent the liquid phase composition of a suspension equilibrating in a triphasic domain.

Right: projection of solubility surface of A (green) on solvent-free surface ABC in the isothermal quaternary phase diagram A/B/C/Solvent. Dotted lines connect solubility compositions (4, a, c, d) to their solvent-free equivalent (4', a', c', d'). The data from the AB axis of the ternary phase diagram on the left are shown on the AB axis. Dashed lines represent the boundaries of the phase stability domain. The solubility surface of A is limited by solubility curves from ternaries A/B/Solvent and A/C/Solvent, and eutectic lines linking ternary eutectic compositions a and c (black triangles) to quaternary composition d (black square) involving A. Quaternary composition d represents the liquid phase composition of a suspension equilibrating in a quadriphasic domain.

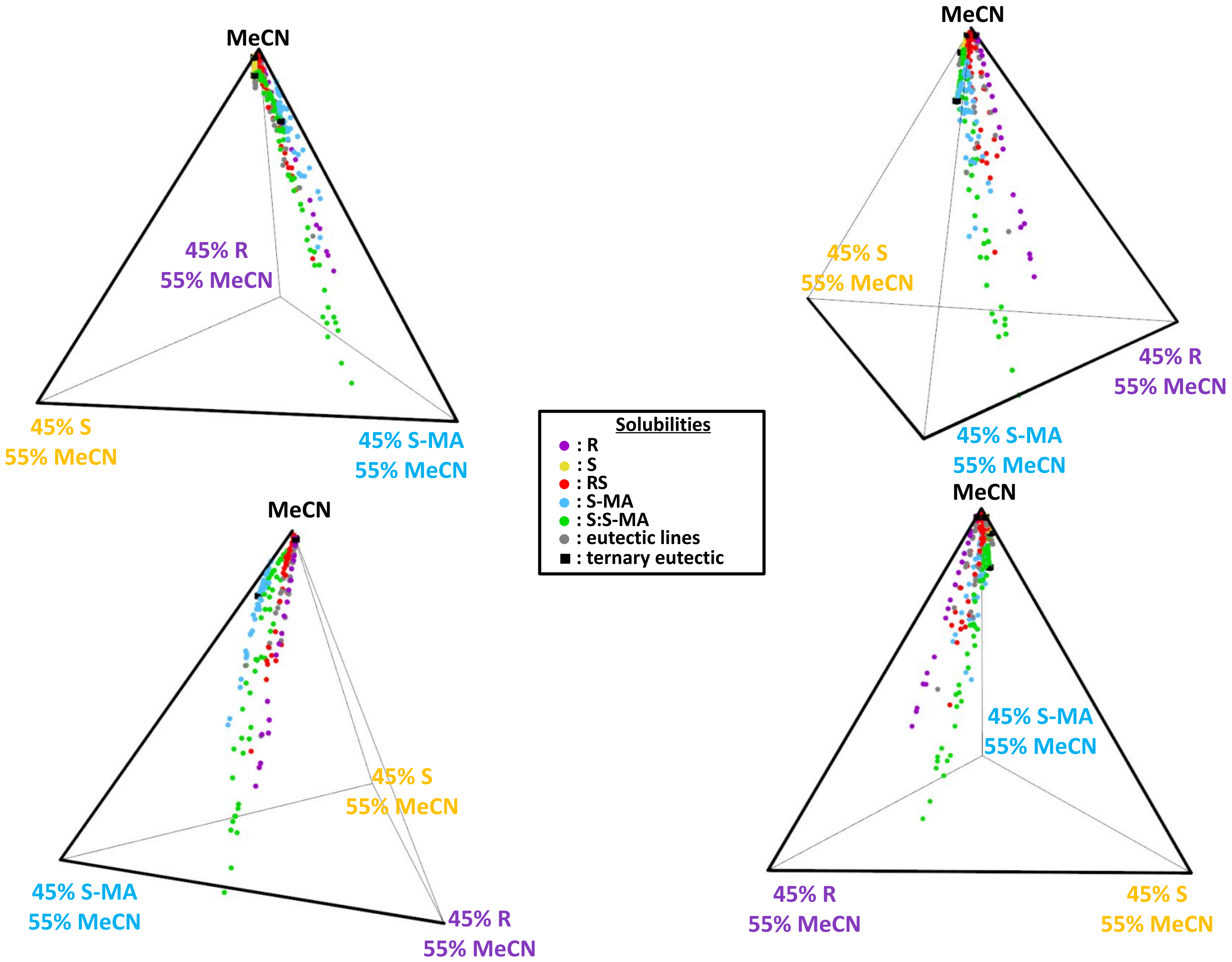

Figure S4: Zoom in the 55% MeCN to 100% MeCN tetrahedron region of the R/S/S-MA/MeCN quaternary phase diagram, with different viewing angles.

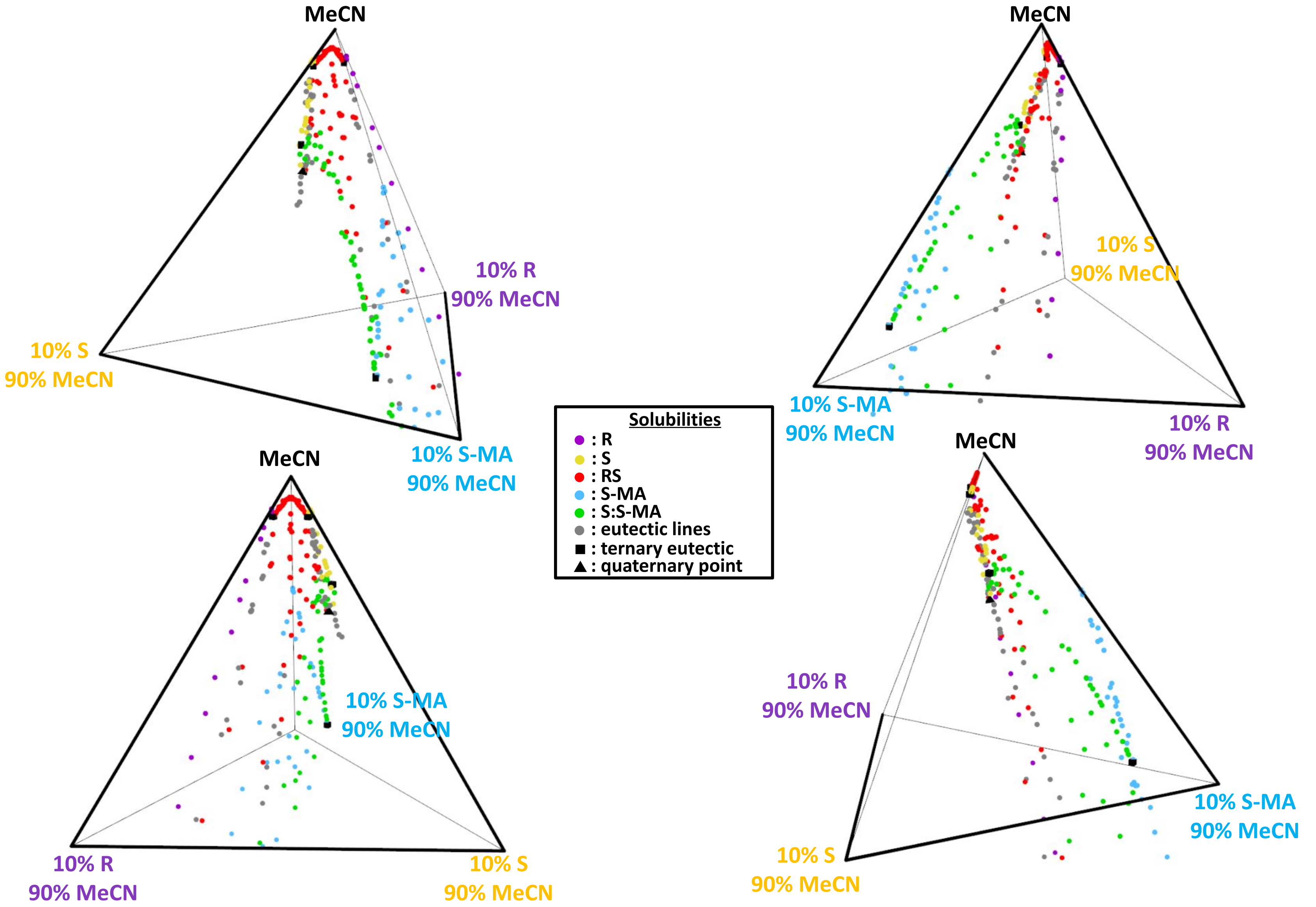

Figure S5: Zoom in the 90% MeCN to 100% MeCN tetrahedron region of the R/S/S-MA/MeCN quaternary phase diagram at 9°C, with different viewing angles.
